# Amniotic Fluid Derived Stem Cells with a Renal Progenitor Phenotype Inhibit Interstitial Fibrosis in Renal Ischemia and Reperfusion Injury in Rats

**DOI:** 10.1371/journal.pone.0136145

**Published:** 2015-08-21

**Authors:** Marina Gabriela Monteiro Carvalho Mori da Cunha, Silvia Zia, Fanny Oliveira Arcolino, Marianne Sylvia Carlon, Diego Vilibaldo Beckmann, Ney Luis Pippi, Dominguita Luhers Graça, Elena Levtchenko, Jan Deprest, Jaan Toelen

**Affiliations:** 1 Department of Development and Regeneration, Organ System Cluster, Fetal therapy group, Group Biomedical Sciences, KU Leuven, Leuven, Belgium; 2 Experimental Veterinary Surgery Laboratory, Universidade Federal de Santa Maria, Santa Maria, Brazil; 3 Department of Development and Regeneration, Organ System Cluster, Laboratory of Pediatric Nephrology, Group Biomedical Sciences, KU Leuven, Leuven, Belgium; 4 Department of Pharmaceutical and Pharmacological Sciences, Laboratory of Molecular Virology and Gene Therapy, Group Biomedical Sciences, KU Leuven, Leuven, Belgium; 5 Department of Obstetrics and Gynecology, University Hospitals Leuven, Leuven, Belgium; 6 Department of Pediatrics, University Hospitals Leuven, Leuven, Belgium; Center for Molecular Biotechnology, ITALY

## Abstract

**Objectives:**

Mesenchymal stem cells derived from human amniotic fluid (hAFSCs) are a promising source for cellular therapy, especially for renal disorders, as a subpopulation is derived from the fetal urinary tract. The purpose of this study was to evaluate if hAFSCs with a renal progenitor phenotype demonstrate a nephroprotective effect in acute ischemia reperfusion (I/R) model and prevent late stage fibrosis.

**Methods:**

A total of 45 male 12-wk-old Wistar rats were divided into three equal groups;: rats subjected to I/R injury and treated with Chang Medium, rats subjected to I/R injury and treated with hAFSCs and sham-operated animals. In the first part of this study, hAFSCs that highly expressed CD24, CD117, SIX2 and PAX2 were isolated and characterized. In the second part, renal I/R injury was induced in male rats and cellular treatment was performed 6 hours later via arterial injection. Functional and histological analyses were performed 24 hours, 48 hours and 2 months after treatment using serum creatinine, urine protein to creatinine ratio, inflammatory and regeneration markers and histomorphometric analysis of the kidney. Statistical analysis was performed by analysis of variance followed by the Tukey’s test for multiple comparisons or by nonparametric Kruskal-Wallis followed by Dunn. Statistical significance level was defined as *p <0*.05.

**Results:**

hAFSCs treatment resulted in significantly reduced serum creatinine level at 24 hours, less tubular necrosis, less hyaline cast formation, higher proliferation index, less inflammatory cell infiltration and less myofibroblasts at 48h. The treated group had less fibrosis and proteinuria at 2 months after injury.

**Conclusion:**

hAFSCs contain a renal progenitor cell subpopulation that has a nephroprotective effect when delivered intra-arterially in rats with renal I/R injury, and reduces interstitial fibrosis on long term follow-up.

## Introduction

Acute kidney injury (AKI) is defined as a rapid and progressive decrease in glomerular filtration rate following an insult, which leads to a loss of renal function [1, 2]. It is a pathology that affects neonatal to geriatric patients, present in 2% of all hospitalized patients [3] and up to two thirds of patients in an ICU setting [4]. Ischemia-reperfusion (I/R) injury remains one of the leading causes of AKI [5, 6]. Despite current clinical treatment for AKI, some patients remain dialysis-dependent or develop progressive chronic kidney disease (CKD) necessitating transplantation [7, 8]. Moreover, I/R injury is an inevitable consequence of kidney transplantation and can induce delayed graft function [9].

The pathophysiology of I/R injury is complex and mainly characterized by proximal tubular cell damage, loss and inflammation, leading to tissue injury and dysfunction. Prolonged ischemia is one of the major contributors to the development of primary kidney graft failure, delayed graft function and enhanced immunogenicity in kidney transplantation, and has a major impact on long-term outcome [10–12]. I/R may enhance the expression of MHC molecules in renal epithelium and could thereby trigger allogeneic acute/chronic rejection [13]. Given the shortage of donor organs and usage of marginal donor kidneys for transplantation, novel treatment options to minimize renal I/R injury are needed [14].

Stem cell-based therapy is a promising approach for kidney regeneration. Mesenchymal stem cells (MSCs) possess the ability to home to areas of tissue injury due to the local production of growth factors and cytokines. An extensive review of the working mechanisms of stem cells lies beyond the scope of this introduction. Briefly, these cells have been shown to influence angiogenesis [15], to have immunomodulatory properties [16], to activate the endogenous progenitors and to promote cellular reprogramming [17]. Additionally, stem cells have also anti-fibrotic, anti-oxidant, anti-inflammatory [18, 19] and anti-apoptotic properties [20, 21].

Several groups have successfully used stem cell therapies in the treatment of AKI in animal models [15, 20, 22–25]. Administration of MSC has resulted in functional recovery following cytotoxic [22, 26] and ischemic AKI [16, 24]. MSCs are also known to attenuate acute graft rejection in the transplanted kidney potentially by modulating reperfusion injury [27–29]. Different tissue sources of SC for the treatment or prevention of renal failure have been investigated including bone marrow [24], adipose tissue [19], cord blood [30] and Wharton’s jelly [31, 32]. Recently, amniotic fluid stem cells (AFSCs) have been investigated for their regenerative potential [33–40]. AFSCs are fetally derived cells with a broader differentiation capacity than adult stem cells and higher proliferation rates [33] and they are less likely to have acquired genetic mutations [41]. Previous studies have shown that amniotic fluid contains a subpopulation of cells that primarily express renal progenitor markers and are able to differentiate into podocytes *in vitro* [42, 43].

The aim of this study was to evaluate whether amniotic fluid derived stem cells with a renal progenitor phenotype have a nephroprotective effect in I/R injury and can prevent renal fibrosis as a late consequence.

## Materials and Methods

### Isolation, expansion and characterization of stem cells from amniotic fluid

The Ethics Committee of the UZ Leuven approved the research program on the regenerative potential of amniotic fluid derived cells isolated from discarded amniotic fluid obtained after clinically indicated amniocentesis procedures. Consenting women in this study were between 15–22 weeks of pregnancy. Cell lines were excluded retrospectively when infection or genetic abnormalities were detected at a later stage. Briefly, amniotic fluid was filtered with a 40 μm strainer (BD bioscience, Erembodegem, Belgium) and centrifuged. The cell pellet was re-suspended in expansion medium consisting of α-MEM (Invitrogen, Ghent, Belgium), 15% fetal bovine serum (Invitrogen, Ghent, Belgium), 1% L-glutamine (Invitrogen, Ghent, Belgium), 1% penicillin/streptomycin (Invitrogen, Ghent, Belgium) and 18% Chang B and 2% Chang C (Irvine Scientific, Brussels, Belgium), plated in a petri dish and incubated at 37°C in 5% CO_2_. As soon as single cells attached to the dish, medium was replaced to remove debris and unwanted epithelial cells. All individual cells were monitored daily using a light microscope. After a few days, single colonies were mechanically transferred into a 96 well plate and expanded as monoclonal populations [44].

For flow cytometry, 100,000 hAFSCs at passage 4 were stained for mesenchymal markers CD117, CD44, CD73, HLA-ABC, CD24 (BD bioscience, Erembodegem, Belgium), CD29 (Acris, Herford, Germany), and the hematopoietic markers CD34 and CD45 (BD bioscience, Erembodegem, Belgium) (see Table 1). Cells were washed three times with PBS and 1% BSA and then incubated with primary antibodies for 20 minutes at 4°C in the dark. Cells were then washed 3 times with PBS and then re-suspended in 200 μl of PBS. Cells were incubated with respective isotype controls and unstained cells were used to define reading settings. Analysis was performed with the FACS Canto (BD bioscience, Erembodegem, Belgium).

**Table 1:** List of Antibodies used for flow cytometry and immunofluorescence analysis.

Protein	Catalog number	Brand
Mouse IgG1-FITC	345815	BD bioscience
Mouse IgG1-PE	345816	BD bioscience
Mouse Anti human CD105-PE	FAB1097P	R&D system
Mouse Anti human CD45-PE	FAB1430P	R&D system
Mouse Anti human HLA-DR-PE	555561	BD bioscience
Mouse Anti human CD117-PE	130-091-734	MACS
Mouse Anti human CD24-FITC	555427	BD bioscience
Mouse Anti human CD73-PE	550257	BD bioscience
Mouse Anti human CD44-PE	550989	BD bioscience
Mouse Anti human HLA-ABC-FITC	560169	BD bioscience
Mouse Anti human SIX2 (monoclonal antibody clone 3D7)	H00010736-M01	Abnova
Rabbit Anti human PAX2 (monoclonal antibody clone 3C7)	ab38738	Abcam
AlexaFluor 488 goat anti-mouse	A-11001	Life Technologies
AlexaFluor 594 goat anti-rabbit	A-11012	Life Technologies
Alexa Fluor 568 donkey anti-goat	A-11057	Life Technologies
Goat anti Mouse IgG-PE	405307	Biolegend
Donkey anti Rabbit IgG-FITC	406403	Biolegend

Differentiation protocols (were performed as previously described [44]):

#### Osteogenic differentiation

Osteogenic differentiation was induced by culturing hAFSCs at 70% confluence for 4 weeks in ‘osteogenic differentiation medium’ (Invitrogen). Differentiation was assessed by Alizarin staining (Sigma Aldrich, Diegem, Belgium) of the calcified extracellular matrix deposition.

#### Adipogenic differentiation

To induce adipogenic differentiation, cells were cultured at 100% confluence and subsequently differentiated with adipogenic differentiation medium for 14 days. This medium was composed of 10% FBS, 10^6^ M dexamethasone, 0.5 M 3-isobutyl-methylxanthine, 10 mg/mL insulin, and 200 mM indomethacin in Dulbecco’s Modified Eagle Medium high glucose (Invitrogen). Differentiation into the adipogenic lineage was determined by Oil Red O staining (Sigma-Aldrich, MO, USA).

#### Chondrogenic differentiation

To induce chondrogenic differentiation, cells were cultured in high-density pellet mass cultures for 14 days. 20 mL droplets of cells suspension (400,000 cells resuspended in PBS) were seeded into individual wells of a 24-well plate. Cells were allowed to attach without medium for 3 h at 37°C in a 5% CO2 incubator and then cultured for 24h in growth medium. 24h later, the medium was replaced with chondrogenic differentiation medium (Invitrogen). Chondrogenic differentiation was determined by Alcian Blue staining (Sigma-Aldrich).

qPCR was performed for detection of differentiation markers on cells before and after differentiation. Briefly, RNA was extracted using TRIPURE (Roche, Vilvoorde, Belgium) according to the manufacturer’s instructions and cDNA was synthesized from 1μg total RNA using TaqMan Reverse Transcriptase kit (Applied Biosystem, Gent, Belgium). qPCR assay consisted of initial UDG incubation and denaturation (50°C for 2min, 95°C for 2 min) followed by 40 cycles of a 2-step PCR (95°C for 15 sec, 60°C for 45 sec) using primers for the gene of interest, Platinum SyBR Green qPCR supermix-UDG (Invitrogen, Gent, Belgium) and 2μl cDNA. Primers used for amplification are provided in Table 2. GAPDH was used as a housekeeping gene to normalize mRNA levels and results were computed using the Delta Ct (Ct _gene of interest_—Ct _GAPDH_) formula and subsequently normalized to the level before differentiation.

**Table 2:** Human primer sequences of target genes.

Genes	Forward	Reverse
**GAPDH**	TGGTATCGTGGAAGGACTCATGAC	ATGCCAGTGAGCTTCCCGTTCAGC
**OSTEOCALCIN**	GTGCAGCCTTTGTGTCCAA	GCTCACACACCTCCCTCCT
**RUNX2**	CGCATTCCTCATCCCAGTAT	GCCTGGGGTCTGTAATCTGA
**ALP (TNAP)**	GGACATGCAGTACGAGCTGA	GTCAATTCTGCCTCCTTCCA
**SOX9**	TGGAGACTTCTGAACGAGAGC	CGTTCTTCACCGACTTCCTC
**COLLAGEN type2 (α1)**	GGCTTCCATTTCAGCTATGG	AGCTGCTTCGTCCAGATAGG
**PAX2**	CCCAGCGTCTCTTCCATCA	GGCGTTGGGTGGAAAGG
**SIX2**	CTCAAGGCACACTACATCGAG	GTTGTGGCTGTTAGAATTGGA
**KSP**	CTCAGGTTCTCCCTAGTCAAT	AGTCTCGGCTCATCTGTATC

### Identification of stem cells with a renal phenotype

To show the nephrogenic potential of a subtype of hAFSCs, multiple monoclonal populations—isolated from different gestational ages (from 15 until 22 and the 26th)—were analyzed by flow cytometry for CD24 [42] and by qPCR for the renal marker Kidney Specific Protein (KSP) [45–47], as well as for SIX2 and PAX2 (Table 2). cDNA from human embryonic kidney (hEK) from the 19^th^ gestational week was used as a control (hEK RNA was donated by Prof. Paul Winyard, University College London, UK).

Among the 120 hAFSCs populations analyzed, one population was chosen for further experiment *in vitro* and *in vivo*. This population of hAFSC was negative for the hematopoietic marker CD34, had a high proliferative capacity (> 30 passages in culture) and was positive (>50%) for the markers CD24, KSP, SIX2 and PAX2.

For immunofluorescence staining, hAFSCs were seeded on 8-well Lab-Tek Chamber Slide System (Thermo Scientific, Aalst, Belgium) at a density of 30,000 cells/chamber. Cells were fixed with 4% paraformaldehyde for 15 minutes. Subsequently they were permeabilized with 0.1% Triton X-100 (Sigma-Aldrich, Diegem, Belgium) for 5 minutes and blocked in blocking buffer containing 2% BSA, 2% FBS and 0.2% gelatin in PBS for 1 hour. Cells were incubated with primary antibodies: mouse anti-SIX2 (H00010736-M01, monoclonal antibody 3D7, Abnova, Taipei City, Taiwan) and rabbit anti-PAX2 (ab38738, polyclonal antibody, Abcam, Cambridge, UK) in dilutions of 1:100 overnight at 4°C. AlexaFluor 488 goat anti-mouse (A-11001, Life Technologies, Merelbeke, Belgium) and AlexaFluor 594 goat anti-rabbit (A-11012, Life Technologies, Merelbeke, Belgium) secondary antibodies were used (1:500) for 1 hour in the dark at room temperature. DAPI was diluted to 1:1000 in mounting medium. Cells were washed 5 times with PBS between steps. Slides were visualized with a confocal microscope (Zeiss LSM 780, Zaventem, Belgium) using a 20x water immersion objective.

For flow cytometry analysis 100,000 cells were used for each condition. Cells were fixed in 2% PFA for 15 minutes and then permeabilized with 0.1% Triton X-100 (Sigma-Aldrich, Diegem, Belgium) for 15 minutes. Cells were blocked in buffer containing 1% BSA in PBS for 15 minutes on ice and then incubated with primary antibodies for SIX2 and PAX2 (1:100) in permeabilizing solution for 30 minutes on ice. Specific isotypes were used and are listed in Table 1. Secondary Ab were added at a concentration of 1:50 in 0.5% Tween 20 in PBS for 30 minutes. Cells were then washed twice with 1% BSA in PBS and resuspended in 200 μl of PBS and read with the FACS Canto.

### Labelling and transplantation of isolated hAFSCs

To track the cells *in vivo*, a lentiviral vector platform was used to introduce the reporter gene β-Galactosidase (LacZ) into the selected monoclonal population of hAFSCs, as described previously [44]. Briefly, lentiviral vector particles were added to the cell culture with a multiplicity of infection of 10. X-gal staining was performed to investigate the transduction efficiency of lentiviral vector in hAFSCs. Cells were fixed in 25% glutaraldehyde and 37% of methanol for 2 minutes and incubated with X-gal solution (Sigma-Aldrich, Diegem, Belgium) overnight at 37°C. Stained cells were counted using light microscopy and expressed as a ‘positive percentage’ in relation to the total cell population (% positive cells). To study the localization of injected cells *in vivo*, 1x10^6^ LacZ transduced hAFSCs were injected into abdominal aorta 6 hours after ischemia and reperfusion (I/R) injury [48]. Rats were harvested 6 and 24 hours after injection. Kidneys, lungs, heart, spleen and liver were frozen in OCT medium. Cryosections of 5 μm thickness were incubated overnight at 37°C with X-gal solution pH 8.5 (vWR International, Heverlee, Belgium), counterstained with paracarmine and mounted in Mowiol.

### Genetic stability testing of hAFSCs

Chromosomal microarray analysis (CMA) was performed on the cell population selected for the *in vivo* experiment to exclude unbalanced chromosomal aberrations. Cells were trypsinized, centrifuged and cell pellets washed twice in PBS. Genomic DNA was extracted using the DNA mini kit (QIAGEN, Venlo, Netherlands) following the manufacturer’s recommendations, with a final elution volume of 60 μl. 500 ng sample of DNA and sex-mismatched reference DNA were labelled and co-hybridized to an 8x60K chromosomal microarray (Oxford Gene Technology, OGT, Oxford, UK) as previously described [49]. Genomic DNA was labeled in Cy3 or Cy5 for 4 hours using the CytoSure Labelling Kit (Oxford Gene Technology), with no enzyme digestion. Hybridization was performed for 40 hours in a rotator oven (SciGene, CA, USA) at 65°C. Washing of arrays was performed using the Little Dipper (SciGene) with Agilent wash buffer solutions and subsequently dried using acetonitrile. Arrays were scanned using an Agilent microarray scanner at 2-μm resolution, followed by calculation of signal intensities using Feature Extraction software (Agilent Technologies, Dieghem, Belgium). Visualization of results and data analysis were performed using the CytoSure Interpret Software (Oxford Gene Technology), which uses the circular binary segmentation (CBS) algorithm. Genomic coordinates were based on build hg19. Quality control metrics were also monitored with the CytoSure Interpret software (Oxford Gene Technology).

Selected hAFSCs cell lines were analysed at an early passage in culture (passage 14) and at a late one (passage 33) to exclude acquired genetic aberrations. To exclude lentiviral induced genotoxicity the labeled LacZ-hAFSC cell line was also tested.

### Induction of ischemia reperfusion injury and stem cell injections in the rat model

All procedures involving animals were approved by the Local Ethics Committee for animal experimentation of the Catholic University of Leuven (KU Leuven) (project number: 088/2013). Animals were housed at constant temperature and humidity, with 12:12-h light-dark cycles, and unrestricted access to standard diet and water. All experiments were performed in cohorts of adult male Wistar rats, 3 months old, weighing 200–300g. Rats (n = 10/group) were randomly assigned as follows: rats subjected to I/R injury and treated with fresh expansion media (= group I/R+ vehicle); rats subjected to I/R injury and treated with hAFSCs (= group I/R + hAFSC) and sham-operated animals (= group no injury). The experimental time line is shown in S1 Fig.

I/R was induced in isoflurane-anesthetized rats as described previously [48]. After a midline laparotomy, the renal pedicles were located and isolated by blunt dissection. Renal pedicles were clamped with atraumatic vascular clamps for 50 min. Kidney reperfusion was confirmed visually after the clamps were removed. The timing of ischemia was optimized to obtain a reversible model of ischemic AKI with a minimum of vascular thrombosis without animal mortality. At six hours after I/R injury, 800 μl of cell suspension (1x10^6^ cells) in fresh expansion media was administered intra-arterially in two steps. Briefly, the abdominal aorta was caudally dissected around the level of the renal arteries. In order to direct the flow of the injected cells towards the kidney, the aorta was occluded with a vascular clamp 1 cm caudal of the renal arteries and veins. Subsequently, the right vascular pedicle was isolated and both the right renal artery and vein were temporarily clamped for 10–20 seconds before injection of half the volume of cells. After injection of the initial 400 μL, the clamp was released and placed on the left renal pedicle, followed by injection of the remaining 400 μL. Fresh expansion media was injected in vehicle group rats using the same method. The operators were blinded to the actual administered products. A sham or ‘no injury group’ was operated in identical fashion, except that the renal pedicles were not clamped and no treatment was injected as previously described [50–53]. Peri-operative pain-relief of 0.05 mg/kg IP q12h of buprenorphine was administered for 2 days. The rats received 5 ml saline after the surgery and kept on a heating pad at 37°C until recovery of locomotion.

### Assessment of renal function after I/R injury

Serum creatinine was measured by colorimetric assay (modified Jaffé technique) at 3 different time points: 24 hours, 48 hours and 2 months after I/R injury. Urine samples were collected at 24 hours (n = 5/group), 48 hours (n = 5/group) and 2 months (n = 5/group) and analyzed for creatinine concentration and urine protein-to-creatinine ratio determination. Total protein concentration was determined by silver staining according to the modified method of Heukeshoven and Dernick [54]. Each urine sample was diluted in water to normalize creatinine levels and treated with loading buffer containing 2% β-mercaptoethanol and Laemmli (Sigma-Aldrich, Diegem, Belgium) and boiled for 5 minutes at 100°C. Protein fractions were separated in a 10% SDS polyacrylamide gel by electrophoresis followed by silver staining. Gels were soaked in 50% methanol, 12% acetic acid, 0.1% formaldehyde and 0.1% glutaraldehyde solution for 45 minutes and then washed 3 times in 50% of methanol for 5, 10 and 15 minutes. Subsequently they were soaked for 45 seconds in 0.2g/L Na_2_S_2_O_3_ and washed 3 times in distilled water. Finally, gels were stained in 2g/L silver nitrate for 20 minutes and washed in distilled water. Developing solution was composed of 60g/L Na_2_CO_3_ and 0.1% formaldehyde. All products were obtained from Sigma-Aldrich, Belgium.

### Histology and immunohistochemistry of renal tissue

Forty-eight hours and 2 months after I/R injury, rats were euthanized and the kidneys were perfused with saline to remove blood from the vascular beds. The kidney specimens were fixed in 4% neutral formalin and embedded into paraffin and then sectioned to 4 μm slides and processed for periodic acid-Schiff (PAS), Masson’s Trichrome, Picrosirius red and immunostaining. All slides were evaluated by experienced renal pathologists (D.L.G) who were blinded to the sample identification. Images were analyzed qualitatively (scores) by independent observers and quantitatively using the ImageJ software. The tubular necrosis, cast and fibrosis scoring system ranged from 0 to 5+ (0, no changes; 1+, very occasional tubular profiles (usually <3/section and <2/HPF) affected by lesion; 2+, more evident lesions affecting 25% of the HPF; 3+, lesions affecting between 25% and 50% of the HPF; 4+, lesions affecting between 50% and 75% of the HPF and 5+, lesion in more than 75% of the HPF).

All morphometric analyses were performed using ImageJ software. Twenty non-overlapping fields on the coronal sections from both kidneys were selected for quantification of acute kidney injury (AKI) at 24 hours, 48 hours and fibrosis at 2 months I/R. PAS stained slides were used for AKI quantification. The number of luminal hyaline casts and cell loss (denudation of the tubular basement membrane) were quantified per field using a 40X objective (high-power field [HPF]). Fibrotic surface area was quantified in the Masson’s trichrome stained slides at 2 months after I/R injury. The digital color images were segmented (color deconvolution plugin) and further binarized in order to measure the percentage of the area stained in blue.

Immunostaining was performed on paraffin sections. After deparaffinization, endogenous peroxidase activity was blocked with 0.5% H_2_O_2_ in PBS for 20 min at room temperature. Sections were then heated at 98°C for 1 hour in citrate buffer (10mmol/L, pH 6) to enhance antigen retrieval. Non-specific binding was minimized by incubating sections in 1% BSA and 2% milk in PBS-0.1% tween 80 for 30 min. Sections were then incubated overnight at 4°C with the primary polyclonal antibodies against α-smooth muscle actin at 1:100 dilution (α-SMA- clone 1A4, DAKO, Heverlee, Belgium), Ki67 at 1:50 dilution (ki-67 clone MIB-5, DAKO, Heverlee, Belgium) and CD68 at 1:200 (ED-1, ab31630, Abcam, Cambridge, UK). Negative controls included buffer alone. Specific labeling was detected with EnVision/HRP Detection Kit (DAKO, Heverlee, Belgium). The color reaction was developed with 3,3'-diaminobenzidine (Sigma-Aldrich, Diegem, Belgium) and sections were counterstained with Mayer hematoxylin. Sections were then dehydrated through graded ethanol, cleared in xylene, and mounted in dePex (BDH, vWR international, Belgium). Immunostained slides were quantified using ImageJ software. Twenty non-overlapping fields on the coronal section from both kidneys were selected for quantification. For CD68 and α-SMA images were assessed at 200X magnification. While for Ki67 expression, positive tubular cells were quantitatively assessed at 400X magnification. The digital color images were processed and analyzed as described above.

### Molecular analysis of inflammation and injury/toxicity

Kidney samples harvested at 48 hours were snap frozen in liquid nitrogen and total RNA and cDNA synthesis were performed as described above. The list of primers used to detect mRNA expression are listed in Table 3. Three different reference genes were tested to find the most stable between biological groups (*gapdh*, *pdhb* and *sdha*). Gen Norm software was used to ascertain the most stable gene between groups. The geometric mean of *pdhb* and *sdha* were used to normalize mRNA level. *Tgf-β1*,*Tnf-α*, *CD204* and *CD206* were investigated to assess inflammation, *eNOS* and *iNOS* to assess oxidative stress, *caspase-3* to quantify apoptosis and *Kim1* to quantify injury and toxicity level in the samples. Results were computed using the Delta Ct (Ct _gene of interest_—Ct _GAPDH_) formula and subsequently normalized to the level of the “No injury” group.

**Table 3:** Rat primer sequences of target genes.

GENE	Forward	Reverse
**Gapdh**	TCAACAGCAACTCCCATTC	CCTGTTGCTGTAGCCATATT
**Pdhb**	GTCTGATGGTCCGCAGATTTAT	CCAGTGGTGATGCTAGAGAATG
**Sdha**	CTTTCCTACCCGCTCACATAC	AGTCCTGCTAAACGGCATAC
**Kim1**	GATGGGCTCTCTGAGCTTTG	AATCTCCCAGGAGCTGGAAT
**Caspase 3**	CCGACTTCCTGTATGCTTACTC	CCAGGGAGAAGGACTCAAATTC
**Tgf-β**	TGAACCAAGGAGACGGAATACAGG	GAGGAGCAGGAAGGGTCGGT
**Tnf-α**	AAGGAGGAGAAGTTCCCAAAT G	AGAGAACCTGGGAGTAGATAAGG
**CD204**	GCAACAGGAGGACATCAGTAAG	GAGGCCCTTGAATTAAGGTGATA
**CD206**	GACGGACGAGGAGTTCATTATAC	GTTGGAGAGATAGGCACAGAAG
**eNOS**	GACCCTCACCGATACAACATAC	CATACAGGATAGTCGCCTTCAC
**iNOS**	CTCAGGCTTGGGTCTTGTTAG	TGTTGTTGGGCTGGGAATAG

### Statistics

Results were expressed as mean ± SE. Analysis of variance followed by Tukey’s test for multiple comparisons was used to analyze renal function, proliferation, and morphometric parameters. Renal histology was analyzed by nonparametric Kruskal-Wallis followed by Dunn testing. qPCR data were analyzed by nonparametric t-test. Statistical significance level was defined as *p <0*.05.

## Results

### Selection of hAFSCs with a renal phenotype

**Fig 1:**
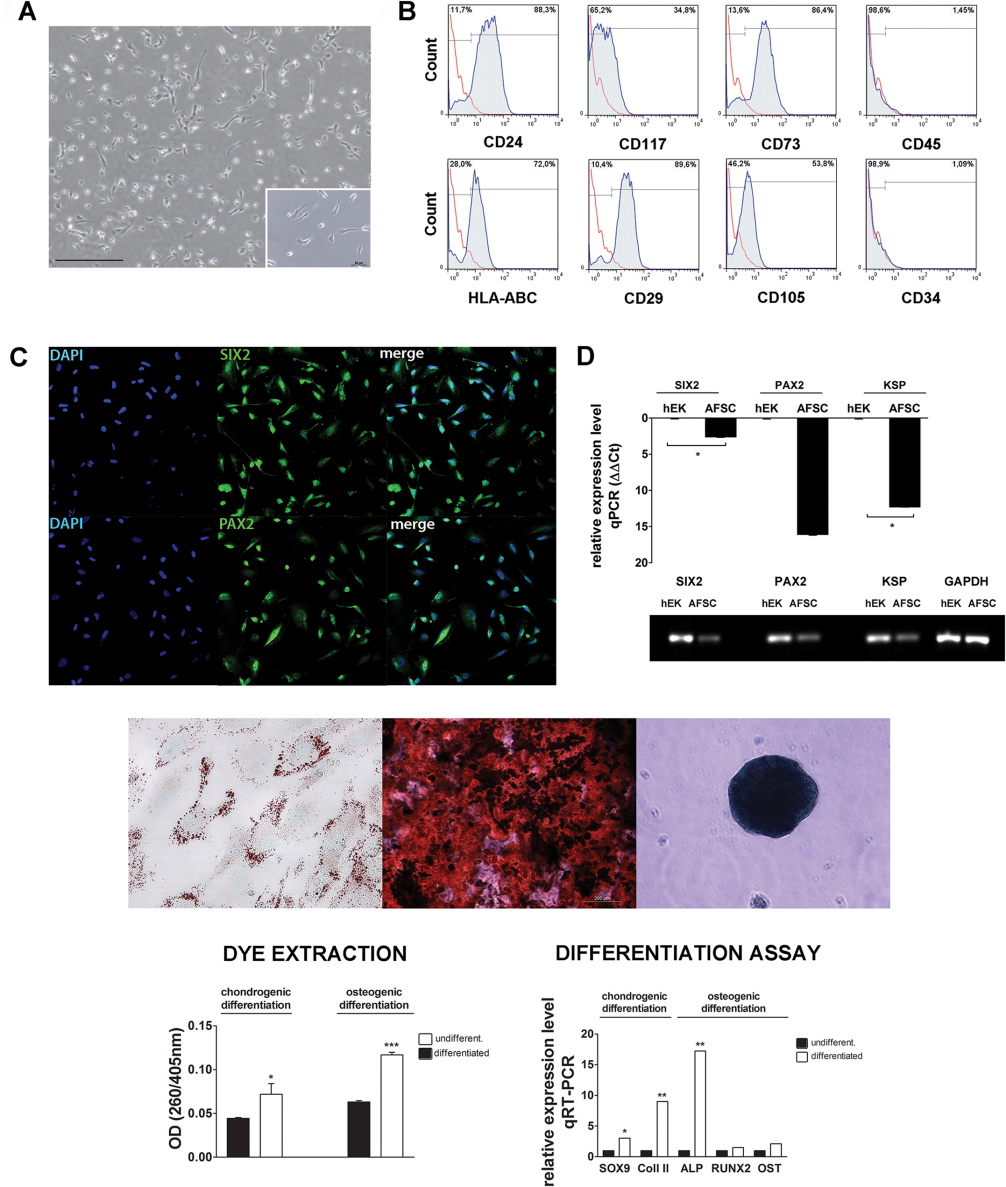
Isolation and characterization of amniotic derived mesenchymal stem cells with renal marker expression (A) Light microscopic image of mesenchymal stem cells isolated from amniotic fluid. A subpopulation of these cells (with renal markers) displays a trumpet shaped phenotype (scale bar = 200 μm). (B) hAFSCs were characterized by flow cytometry (positive for CD24, CD117, CD73, HLA-ABC, CD29, and CD105 but not for CD45, or CD34). (C) Confocal imaging of hAFSCs for renal markers SIX2 and PAX2. (D) Expression of Six2 and Pax2 (left panel) in hAFSCs compared to human embryonic kidney (hEK). Agarose gel analysis of PCR fragments (SIX2: 336 bp; PAX2: 65 bp; KSP: 152bp; GAPDH: 189 bp) (E). hAFSCs were differentiated along adipogenic, osteogenic and chondrogenic lineages (left to right). In the adipogenic differentiation the cells formed lipid vesicles stained with oil red O (left) (magnification 100X). Calcium deposits stained with Alizarin confirmed osteogenesis (center). Micromasses stained with alcian blue confirmed chondrogenesis (right) (scale bar = 200 μm). Quantification of chondrogenic and osteogenic differentiation using dye extraction and quantification with spectrophotometry before and after differentiation (left panel). qPCR analysis of chondrogenic markers (Sox9, Col II) and osteogenic markers (ALP, Runx2 and OST) before and after differentiation (right panel).

Stemness characteristics of hAFSCs were proven by differentiation into adipogenic, osteogenic and chondrogenic lineages as depicted in Fig 1E. Differentiation was quantified by dye extraction (Fig 1D, right panel) and by qPCR analysis. Upon chondrogenic differentiation, there was an induction of Sox9 (3-fold), an important gene in the early phase of chondrogenesis and of Collagen II (9-fold), a major protein component of cartilage [55]. Upon osteogenic differentiation there was minor increase in Runx2 and Osteocalcin while ALP expression was significantly increased (17-fold), which suggests that hAFSCs started the deposition of matrix proteins [56] (Fig 1C, middle panel).

61% of the isolated hAFSCs populations expressed the markers KSP, SIX2 and PAX2 (S2 Fig). hAFSCs were morphologically uniform and showed a characteristic trumpet shape (Fig 1A, insert). The selected population expressed MSCs markers and was negative for the hematopoietic ones (Fig 1B). The monoclonal hAFSCs population used for the *in vitro* and *in vivo* experiments, expressed SIX2 and PAX2 by immunofluorescence staining (Fig 1C, left panel) and by qPCR (Fig 1D). 94% of the cells were double positive for SIX2 and PAX2 as shown by flow cytometry data (S3 Fig). Cells negative for SIX2 and PAX2 showed a slightly larger cytoplasm compared to positive cells as noted in the IF staining (S3 Fig).

### Labelling of hAFSCs for *in vivo* tracking

**Fig 2:**
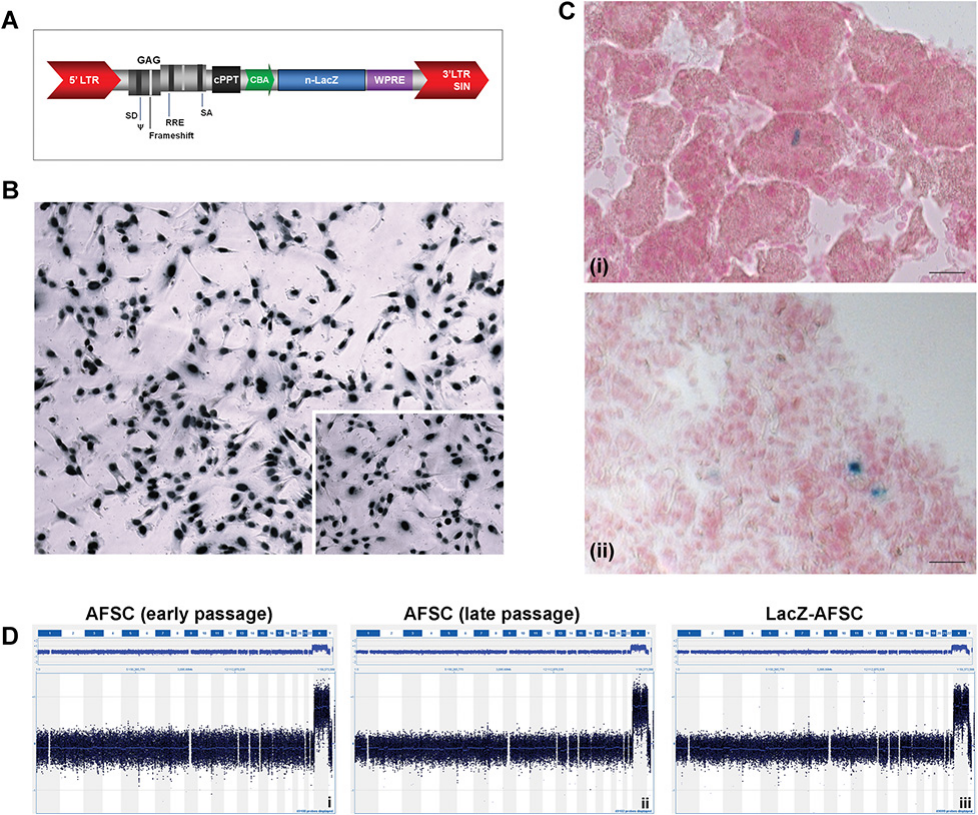
Labelling, in vivo tracking and genomic stability testing of amniotic fluid derived renal stem cells. (A) Schematic representation of the lentiviral transfer construct. The nuclear localized LacZ reporter gene (n-LacZ) is driven by the chicken beta-actin promoter (CBA) and is followed by the woodchuck hepatitis post-transcriptional regulatory element (WPRE). The promoter is preceded by the central polypurine tract (cPPT) and the LV cassette is flanked by the 5′ long terminal repeat (LTR) and 3′ self-inactivating (SIN) LTR. (B) *In vitro* β-galactosidase staining after stable transduction. (C) *In vivo* engraftment of n-LacZ-cells with a representative figure of the kidney after 6h and the lung after 24h. This image does not suggest engraftment of the cells in the target organ, merely their presence. (D) CGH dataset displays the 60K genome-wide array results for a hAFSCs cell line with renal phenotype at passage 14 (left), at passage 30 (middle) and after lentiviral manipulation (right).

hAFSCs were efficiently transduced by the lentiviral vector platform (Fig 2A) as shown by the X-gal staining, which detected the nuclear activity of β-galactosidase in >95% of the cells (Fig 2B). These labeled hAFSCs maintained their mesenchymal profile when analyzed by flow cytometry (data not shown) and were still positive for nuclear X-gal staining after 20 passages in culture. Lac-Z labeled hAFSCs were tracked in both kidneys and lungs at 6 hours after injection (Fig 2C) and only in the lungs and spleen at 24 hours after injection (data not shown). Tracking of cells at later time points (48h and 2m) did not reveal the presence of injected hAFSCs in the renal tissue (data not shown).

The selected cell population was analyzed for the occurrence of spontaneous chromosomal abnormalities or lentiviral mediated genotoxicity (Fig 2D). The imbalances observed for the X and Y chromosomes are due to sex-mismatch of the reference DNA sample which acts as an internal experimental control. No abnormal molecular karyotypes were detected in early passage in culture (passage 14) nor after expansion (passage 33). Lentiviral manipulation (Fig 2D) did not affect karyotyping stability.

### Protective effect of stem cells in experimental renal I/R injury

**Fig 3:**
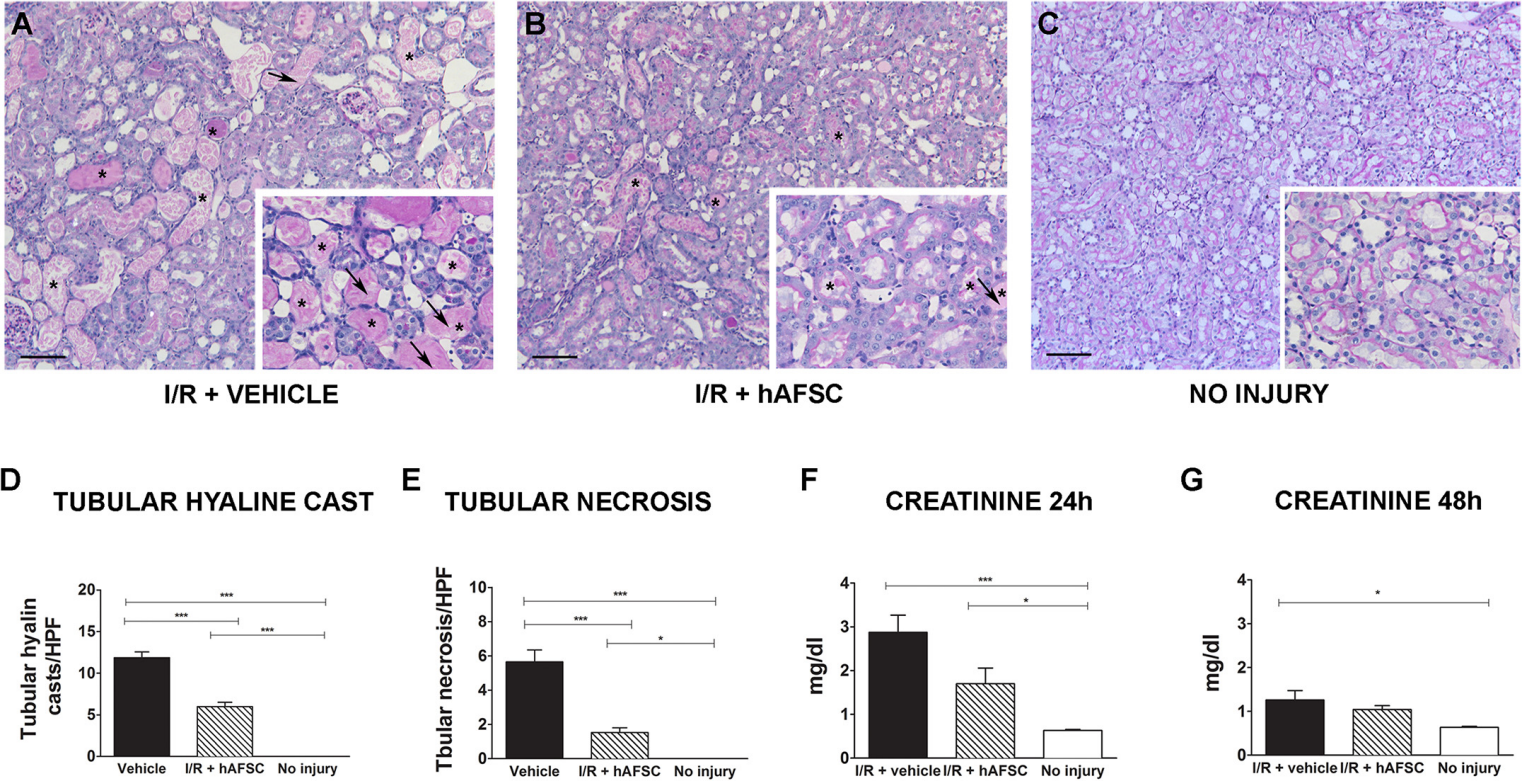
Protective effect of stem cell therapy on acute kidney ischemia reperfusion injury. PAS staining of representative kidney sections from different experimental groups. (A) The epithelial injury was characterized by tubular necrosis (shown by arrows), intratubular hyaline casts (shown by asterisk *), denudation of basal membrane and vacuolization of tubular epithelial cells with a loss of their brush border. (B) The renal tissue showed less severe injury, with less hyaline casts and tubular necrosis in the group treated with stem cells (arrow indicates presence of brush borders). (C) Histology of a kidney without injury (D). Quantitative analysis of the presence of tubular hyaline casts, (E) tubular necrosis (F-G) and serum creatinine levels (mg/dl) at 24h and at 48h of the three cohorts (vehicle treated (*black)*, hAFSC treated (*striped)* and no injury (*white)*). * p<0.05, ** p<0.01 *** p<0.001. (scale bar = 50 μm)

To evaluate the effect of hAFSCs on AKI, we used a surgical model to induce ischemia/reperfusion damage in male Wistar rats [48]. Histological analysis of the kidneys of animals at 48h after injury showed proximal tubular injury characterized by tubular necrosis, denudation of the basal membranes and vacuolization of tubular epithelial cells (Fig 3A). In addition, proximal tubular cells showed a loss of brush borders and the presence of intratubular hyaline casts (Fig 3). The morphometric evaluation of AKI in the cohort treated with hAFSCs revealed a significantly lower number of hyaline cast-containing tubules (5.99 vs 11.87 cast/HPF, p<0.0001) and a lower number of necrotic tubules (1.53 vs 5.65 tubular necrosis/HPF, p<0.05) as compared with I/R + vehicle group. These results were similar to the ATN scores reported by the pathologist (S4B Fig). Similar morphometric results were observed at 24h after injury (S2C Fig). The renal function, as measured by serum creatinine level, was impaired in the short term after I/R. The serum creatinine level in rats treated with hAFSC were significantly reduced at 24h, compared to animals treated with vehicle alone (1.7 versus 2.8 mg/dl, p = 0.0002). In addition, treatment with stem cells resulted in the normalization of the creatinine levels at 48h, whereas controls still had an increased creatinine level compared to uninjured animals (1.0 versus 1.2 mg/dl, p = 0.0412) (Fig 3F and 3G).

### Effect of hAFSCs on tubular proliferation, inflammation and myofibroblast formation

**Fig 4:**
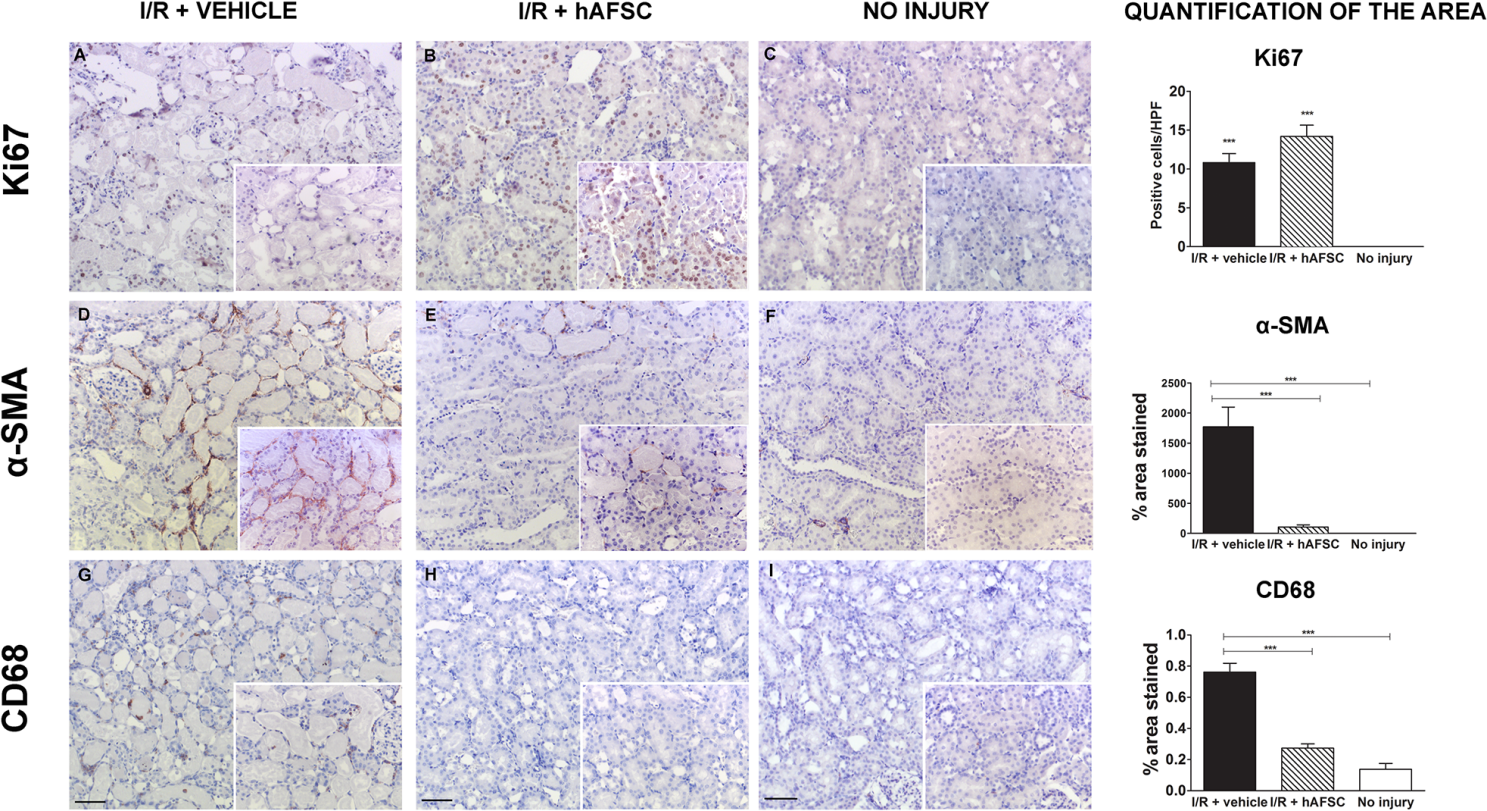
Enhancement of cell proliferation and decrease of myofibroblast differentiation, inflammation and fibrosis after stem cell therapy on acute kidney ischemia and reperfusion injury. Immunohistochemical staining of representative kidney sections from different experimental groups for proliferation (Ki67, Panel A-C), myofibroblast differentiation (α-SMA, Panel D-F) and macrophage infiltration (CD68, Panel G-I) at 48 hours after injury (scale bar = 20 μm). Quantitative analysis of the presence of the representative markers are depicted on the right for the three cohorts (vehicle treated (*black)*, hAFSC treated (*striped)* and no injury (*white)*). * p<0.05, ** p<0.01, *** p<0.001 (scale bar = 50 μm).

To assess the regenerative response of kidney tissue in stem cells injected animals, we quantified Ki-67, CD68 and α-SMA expression at 48 hours after injury (Fig 4). hAFSCs enhanced the proliferation rate of tubular cells after I/R injury as detected by Ki67 expression compared to vehicle treated controls (14.2 vs 10.8, not significant) (Fig 4A–4C). Animals with no injury had negligible Ki67 expression in renal tissue. Moreover, in comparison with non-injured animals, I/R injury induced a massive infiltration of macrophages and an increased presence of myofibroblasts in the kidneys (Fig 4D–4I). A significant decrease of macrophage infiltration and myofibroblast formation was observed in rats treated with stem cells (0.76 vs 0.27, p< 0.0001) and (1771.63 vs 105.548, p<0.0001) respectively. The injury level quantified by qPCR showed an increased expression of Kim1 and Caspase-3 compared to the no-injury group. No statistical difference between treated groups was observed (Kim1: p = 0.9307; Caspase 3: p = 0.6623). To quantify the M1-type macrophage infiltration, iNOS and Tnf-α were assessed, whereas CD 206 and 204 were used to measure the presence of M2-type macrophages. Animals treated with hAFSC showed a significant down-regulation of iNOS, but not Tnf-α expression (iNOS: p = 0.0286; Tnf-α: p = 0.8413), nor did we observe any difference in the expression of mannose receptor (CD206) and scavenger receptor (CD204) (CD206: p = 0.9048; CD204: p = 0.1905). There was no difference in expression of Tgf-β1 (Tgf-β1: p = 0.1255) or eNOS (p = 0.1905) (S5 Fig).

### Inhibition of the progression to chronic kidney disease

**Fig 5:**
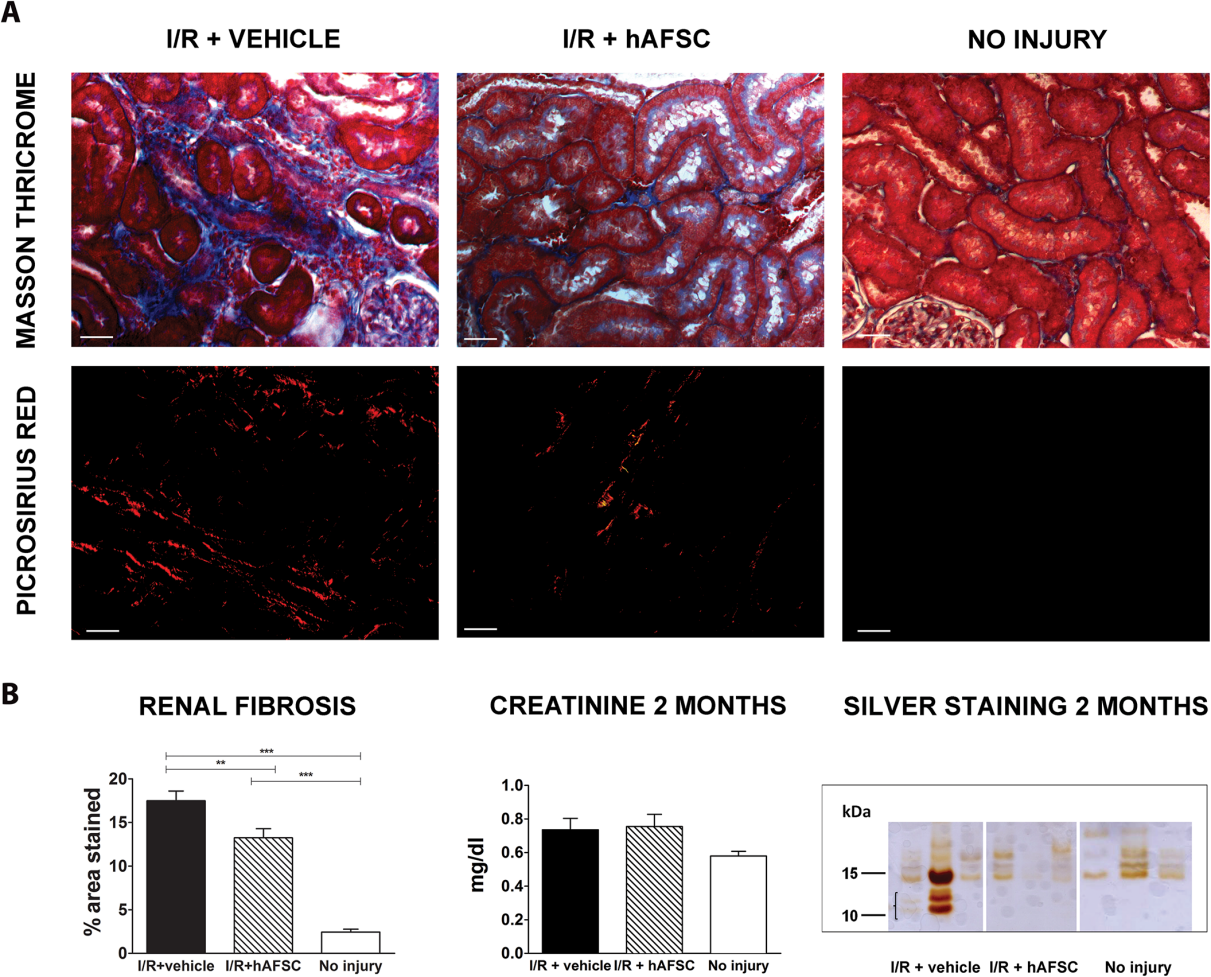
Long-term preservation of renal function and inhibition of tubulo-interstitial fibrosis induced by stem cell therapy. (A) Histochemical staining of representative kidney sections from different experimental groups using Masson’s and PicroSirius at 2 months after injury for the three cohorts. (B) Quantitative analysis of the presence of renal fibrosis depicted on the left for the three cohorts (vehicle treated (*black)*, hAFSC treated (*striped)* and no injury (*white)*). The middle panel shows the serum creatinine values for the three groups (no statistically significant difference). The right panel shows a representative image of a silver staining procedure of urine at 2 months (n = 3 per group). The group with renal reperfusion injury shows the presence of micro-proteinuria, which was absent in both other groups. * p<0.05, ** p<0.01 *** p<0.001. (scale bar = 20 μm)

To assess if the nephroprotective effect observed in the early phase would also result in a reduced fibrogenesis in the long term, interstitial fibrosis was evaluated by Masson’s Trichrome and Picrosirius red staining at 2 months after injury (Fig 5). Our analysis demonstrated significantly less interstitial fibrosis in the hAFSC-treated group compared to I/R+vehicle group (17.49 vs 13.24, p<0.05) (Fig 5A and 5B). These results were in line with fibrosis scores given by the pathologist (S4B Fig). Although there was no significant difference between the creatinine levels of the groups at 2 months (0.736 vs 0.755 mg/dl, p = 0,0616), we could observe an increase in the microproteinuria levels in the group IR+vehicle, while hAFSC-treated rats had urine protein levels comparable to the no injury group as shown by silver staining (Fig 5, bottom right panel). Hence, we observed an inhibitory effect of the stem cells on the progression of CKD after renal I/R injury.

## Discussion

hAFSC represent a more heterogeneous population of stem cells when compared to many adult cell types such as bone marrow or adipose derived stem cells. The fetus sheds cells from the renal, gastrointestinal, dermal, respiratory system and even from the amniotic membranes [57] at different rates and ratios depending on the specific stages of organ development. Isolating stem cells of renal origin will thus depend on the structural and functional development of the renal tract. During nephrogenesis, after the initial contact of the ureteric bud with the metanephric mesenchyme, the condensed mesenchyme generates a population of stem and progenitor cells, which are found at the periphery of the nephrogenic zone in the developing kidney [58]. Such stem-like cells have been demonstrated to be present in the amniotic fluid and they were shown to be able to differentiate *in vitro* into podocytes [43]. Our experiments corroborate these findings. We have identified cells that behave as mesenchymal stem cells based on their expression markers and proliferation and differentiation capacity, but that are also positive for markers that are found in the fetal kidney.

In this study we used cells obtained from standard clinical amniocentesis procedures between 15–22 weeks of gestation. At this stage of the pregnancy a large part of the amniotic fluid consists of fetal urine [59, 60]. In our samples most of the isolated colonies of stem cells at this stage expressed KSP and the renal progenitor markers SIX2 and PAX2. From these specific lineages we selected the clone with the highest proliferative capacity (passage number >30) and the highest expression of CD24, KSP, SIX2 and PAX2 (but not late renal differentiation markers data not shown). It has been shown that AFSCs express mesenchymal markers and differentiate in the mesenchymal lineages (osteogenic, chondrogenic and adipogenic), while at the same time they are able to differentiate into other tissue types (such as neurons,… [33]). In the previous studies, a stem-like cell population derived from human amniotic fluid and possessing characteristics of podocyte precursors were selected based on the expression of CD24 and OB-cadherin [42, 43].

In the second part of our experiments, we investigated the therapeutic potential of these cells in an animal model of AKI. As we intended to locate the injected cells in our animal model, we introduced a reporter gene into our cell line. We opted for a stable genetic introduction as some cell populations are known to release live-cell-dyes [61, 62] and that the transient overexpression of reporter gene decreases after every cell division, which leads to loss of signal. We ensured that the CD marker expression, the differentiation capacity and the karyotypic stability of the cell line remained unchanged after this genetic manipulation. We detected the injected cells in the kidney at 6h after injection but no longer at 24 or 48h. Initial research of MSC based therapy demonstrated their usage as safe with little to no host response [63]. However, the full range of MSC-mediated immune-modulation remains incompletely understood, as emerging reports also reveal that MSCs can adopt an immunogenic phenotype [64]. In our experiments we did not study the possible immune responses of the host towards the injected cells. We did not assume that they would be present for longer periods in a xenograft context with an immunocompetent host. We have chosen an arterial delivery route, as the IA administration of MSCs has been shown to result in a more significant nephroprotection [65–67].

The fact that the cells cannot be detected during later time points in the kidney suggests that hAFSCs are less likely to have an effect on the pathophysiological processes by direct integration into the kidney, but may play a paracrine role during the early phases of the ischemic events.

When we appraised the effect of the cells in the early phase of the ischemic event, we observed nephroprotective effects at 24h as shown by a significantly lower level of serum creatinine in the hAFSCs group compared to the controls. In this sublethal ischemia model the most pronounced increase in creatinine is noted at 24h, with normalization between 3 to 5 days [68], which is in line with the creatinine value at 48h in our study. We next determined the degree of tubular necrosis and tubular cast formation and detected a marked difference between the cohorts treated with hAFSC and the controls both at 24 and 48h. We also investigated the possible pathways by which the cells could exert their protective effects. Previous studies have focused on the proliferation of epithelial cells, inflammation and myofibroblast differentiation in the affected kidneys [69]. We noted a marked increase in the presence of proliferating cells in the renal tissue 48h after the ischemic episode. The levels of Ki67 positive cells were higher in both injured groups, but in the hAFSC cohort this was even higher. This suggests that the hAFSCs exert a part of their effect through stimulating proliferation and modulation of the regenerative processes in the damaged tissue. The presence of macrophages were also evaluated as a marker for the inflammatory response. The injection of stem cells led to a decreased infiltration of M1 macrophages into the ischemic organs as seen by lower expression of CD68 and iNOS. Mesenchymal stem cells are known to induce the switch from M1 to M2 macrophages [70] but no upregulation was seen in our hAFSCs treated cohort. Other research has shown that M2 macrophages are present in the late phases of the repair [71, 72]. Finally we assessed the myofibroblast differentiation in the injured cohorts and noticed that the stem cells exert a negative effect on this process. The lower expression of α-SMA is indicative of inhibition of fibrosis, as myofibroblasts increase the deposition of extracellular matrix (especially type I collagen) leading to glomerular and tubular atrophy [73].

In the final part of this study we investigated the long-term effect of the hAFSCs injection on post-ischemic injury. We quantified the presence of subcapsular and interstitial fibrosis by morphometry. Based on our observations we concluded that hAFSCs significantly decrease fibrotic process in the renal I/R injury model in rats. These data are in line with research by Baulier E. *et al*., who show an inhibition of fibrosis in a porcine model of kidney transplantation treated with AFSCs at 6 days after transplantation [40]. At the functional level we noticed, in the untreated animals, a higher level of proteinuria, which was not present in the hAFSCs treated cohort. This suggests an inhibition of the progression to CKD after renal I/R injury since microproteinuria can indicate early glomerular injury [74].

The renoprotective mechanism of stem cell therapy has not been fully elucidated. Some studies suggest that MSCs are capable to fuse with resident tubular cells [75, 76], others suggest that stem cells are able to differentiate into resident kidney cells [43, 77]. Today the most prominent mechanism of action seems to constitute a paracrine effect [78–80]. Kidney constitutively express eNOS that plays an important role in the vascular homeostasis by promoting vasodilatation [81]. After renal I/R a downregulation of eNOS is observed which can exacerbate the injury by vasoconstriction and endothelial dysfunction. The increase of eNOS expression in the stem cell treated group seems to be one of the possible pathways involved in the nephroprotective effect of hAFSC in the I/R model. Further research will need to elucidate the exact pathways that are responsible for this effect and investigate if AFSC are indeed a promising experimental therapy for the treatment of AKI in men.

## Conclusion

hAFSCs contain a subpopulation of clonal lineage with a renal progenitor phenotype that have a nephroprotective effect when delivered intra-arterially in rats with renal I/R injury, leading to an inhibition of interstitial fibrosis on long term follow up.

## Supporting Information

S1 Fig Schema of experimental setting. (TIF)

S2 Fig Expression of the renal markers KSP, SIX2 and PAX2 in 4 clonal populations of AFSC of different gestational weeks. Expression level is calculated to the expression of these markers in PTEC cell line.
(TIF)

S3 Fig (A) Morphology of hAFSC positive for SIX2 and PAX2 are underlined using a bright field microscope (magnification 20X). (B) SIX2-PAX2 and CD24-CD117 expression by Flow cytometry analysis. (TIF)

S4 Fig (A) Weight of right kidneys harvested at 48 hours and 2 months. (B) Tubular necrosis and fibrosis scores performed in a blinded fashion by a pathologist (* p<0.05, ** p<0.01 *** p<0.001). (C) Quantitative analysis of the presence of tubular hyaline casts and tubular necrosis at 24h of the three cohorts (vehicle treated (*black)*, stem cell treated (*striped)* and no injury (*white)*). * p<0.05, ** p<0.01, *** p<0.001. (TIF)

S5 Fig qPCR data in kidney harvested at 48 hours after I/R injury. Expression level of the markers CD204, CD206, iNOS, eNOS, Kim1, Caspase3, TGFβ1 and TNFα in the three groups. * p<0.05.
(TIF)

## References

[1] Warnock DG. Towards a definition and classification of acute kidney injury. Journal of the American Society of Nephrology: JASN. 2005;16(11):3149–50. 10.1681/ASN.2005090934 . 16207828

[2] Schrier RW, Wang W, Poole B, Mitra A. Acute renal failure: definitions, diagnosis, pathogenesis, and therapy. The Journal of clinical investigation. 2004;114(1):5–14. 10.1172/JCI22353 15232604 PMC437979

[3] Lameire NH, Bagga A, Cruz D, De Maeseneer J, Endre Z, Kellum JA, et al Acute kidney injury: an increasing global concern. Lancet. 2013;382(9887):170–9. 10.1016/S0140-6736(13)60647-9 . 23727171

[4] Singbartl K, Kellum JA. AKI in the ICU: definition, epidemiology, risk stratification, and outcomes. Kidney international. 2012;81(9):819–25. 10.1038/ki.2011.339 . 21975865

[5] Jang HR, Ko GJ, Wasowska BA, Rabb H. The interaction between ischemia-reperfusion and immune responses in the kidney. J Mol Med (Berl). 2009;87(9):859–64. 10.1007/s00109-009-0491-y . 19562316

[6] Giraud S, Favreau F, Chatauret N, Thuillier R, Maiga S, Hauet T. Contribution of large pig for renal ischemia-reperfusion and transplantation studies: the preclinical model. J Biomed Biotechnol. 2011;2011:532127 10.1155/2011/532127 21403881 PMC3051176

[7] Bucaloiu ID, Kirchner HL, Norfolk ER, Hartle JE 2nd, Perkins RM. Increased risk of death and de novo chronic kidney disease following reversible acute kidney injury. Kidney international. 2012;81(5):477–85. 10.1038/ki.2011.405 . 22157656

[8] Wald R, Quinn RR, Adhikari NK, Burns KE, Friedrich JO, Garg AX, et al Risk of chronic dialysis and death following acute kidney injury. Am J Med. 2012;125(6):585–93. 10.1016/j.amjmed.2012.01.016 . 22516564

[9] Salahudeen AK. Consequences of cold ischemic injury of kidneys in clinical transplantation. Journal of investigative medicine: the official publication of the American Federation for Clinical Research. 2004;52(5):296–8. . 15551651 10.1136/jim-52-05-28

[10] Ojo AO, Wolfe RA, Held PJ, Port FK, Schmouder RL. Delayed graft function: risk factors and implications for renal allograft survival. Transplantation. 1997;63(7):968–74. . 9112349 10.1097/00007890-199704150-00011

[11] Pratschke J, Wilhelm MJ, Laskowski I, Kusaka M, Paz D, Tullius SG, et al The influence of donor brain death on long-term function of renal allotransplants in rats. Transplant Proc. 2001;33(1–2):693–4. . 11267022 10.1016/s0041-1345(00)02207-7

[12] Schroppel B, Legendre C. Delayed kidney graft function: from mechanism to translation. Kidney international. 2014;86(2):251–8. 10.1038/ki.2014.18 . 24522494

[13] Kotsch K, Martins PN, Klemz R, Janssen U, Gerstmayer B, Dernier A, et al Heme oxygenase-1 ameliorates ischemia/reperfusion injury by targeting dendritic cell maturation and migration. Antioxid Redox Signal. 2007;9(12):2049–63. 10.1089/ars.2007.1801 . 17854277

[14] Kortekaas KA, de Vries DK, Reinders ME, Lievers E, Ringers J, Lindeman JH, et al Interleukin-9 release from human kidney grafts and its potential protective role in renal ischemia/reperfusion injury. Inflammation research: official journal of the European Histamine Research Society [et al]. 2013;62(1):53–9. 10.1007/s00011-012-0550-7 . 22971662

[15] Chen J, Park HC, Addabbo F, Ni J, Pelger E, Li H, et al Kidney-derived mesenchymal stem cells contribute to vasculogenesis, angiogenesis and endothelial repair. Kidney international. 2008;74(7):879–89. 10.1038/ki.2008.304 18596729 PMC2782525

[16] Togel F, Hu Z, Weiss K, Isaac J, Lange C, Westenfelder C. Administered mesenchymal stem cells protect against ischemic acute renal failure through differentiation-independent mechanisms. American journal of physiology Renal physiology. 2005;289(1):F31–42. 10.1152/ajprenal.00007.2005 . 15713913

[17] Cantaluppi V, Gatti S, Medica D, Figliolini F, Bruno S, Deregibus MC, et al Microvesicles derived from endothelial progenitor cells protect the kidney from ischemia-reperfusion injury by microRNA-dependent reprogramming of resident renal cells. Kidney international. 2012;82(4):412–27. 10.1038/ki.2012.105 . 22495296

[18] Semedo P, Wang PM, Andreucci TH, Cenedeze MA, Teixeira VP, Reis MA, et al Mesenchymal stem cells ameliorate tissue damages triggered by renal ischemia and reperfusion injury. Transplantation proceedings. 2007;39(2):421–3. 10.1016/j.transproceed.2007.01.036 . 17362746

[19] Feng Z, Ting J, Alfonso Z, Strem BM, Fraser JK, Rutenberg J, et al Fresh and cryopreserved, uncultured adipose tissue-derived stem and regenerative cells ameliorate ischemia-reperfusion-induced acute kidney injury. Nephrology, dialysis, transplantation: official publication of the European Dialysis and Transplant Association—European Renal Association. 2010;25(12):3874–84. 10.1093/ndt/gfq603 20921297 PMC2989793

[20] Perin L, Sedrakyan S, Giuliani S, Da Sacco S, Carraro G, Shiri L, et al Protective effect of human amniotic fluid stem cells in an immunodeficient mouse model of acute tubular necrosis. PloS one. 2010;5(2):e9357 10.1371/journal.pone.0009357 20195358 PMC2827539

[21] Yuan L, Wu MJ, Sun HY, Xiong J, Zhang Y, Liu CY, et al VEGF-modified human embryonic mesenchymal stem cell implantation enhances protection against cisplatin-induced acute kidney injury. American journal of physiology Renal physiology. 2011;300(1):F207–18. 10.1152/ajprenal.00073.2010 . 20943766

[22] Morigi M, Introna M, Imberti B, Corna D, Abbate M, Rota C, et al Human bone marrow mesenchymal stem cells accelerate recovery of acute renal injury and prolong survival in mice. Stem cells. 2008;26(8):2075–82. 10.1634/stemcells.2007-0795 . 18499895

[23] Lin F, Cordes K, Li L, Hood L, Couser WG, Shankland SJ, et al Hematopoietic stem cells contribute to the regeneration of renal tubules after renal ischemia-reperfusion injury in mice. Journal of the American Society of Nephrology: JASN. 2003;14(5):1188–99. . 12707389 10.1097/01.asn.0000061595.28546.a0

[24] Duffield JS, Bonventre JV. Kidney tubular epithelium is restored without replacement with bone marrow-derived cells during repair after ischemic injury. Kidney Int. 2005;68(5):1956–61. 10.1111/j.1523-1755.2005.00629.x 16221175 PMC2915580

[25] Bi B, Schmitt R, Israilova M, Nishio H, Cantley LG. Stromal cells protect against acute tubular injury via an endocrine effect. Journal of the American Society of Nephrology: JASN. 2007;18(9):2486–96. 10.1681/ASN.2007020140 . 17656474

[26] Herrera MB, Bussolati B, Bruno S, Fonsato V, Romanazzi GM, Camussi G. Mesenchymal stem cells contribute to the renal repair of acute tubular epithelial injury. International journal of molecular medicine. 2004;14(6):1035–41. . 15547670

[27] Hara Y, Stolk M, Ringe J, Dehne T, Ladhoff J, Kotsch K, et al In vivo effect of bone marrow-derived mesenchymal stem cells in a rat kidney transplantation model with prolonged cold ischemia. Transplant international: official journal of the European Society for Organ Transplantation. 2011;24(11):1112–23. 10.1111/j.1432-2277.2011.01328.x . 21880071

[28] Tan J, Wu W, Xu X, Liao L, Zheng F, Messinger S, et al Induction therapy with autologous mesenchymal stem cells in living-related kidney transplants: a randomized controlled trial. JAMA. 2012;307(11):1169–77. 10.1001/jama.2012.316 . 22436957

[29] Reinders ME, de Fijter JW, Roelofs H, Bajema IM, de Vries DK, Schaapherder AF, et al Autologous bone marrow-derived mesenchymal stromal cells for the treatment of allograft rejection after renal transplantation: results of a phase I study. Stem Cells Transl Med. 2013;2(2):107–11. 10.5966/sctm.2012-0114 23349326 PMC3659754

[30] Morigi M, Rota C, Montemurro T, Montelatici E, Lo Cicero V, Imberti B, et al Life-sparing effect of human cord blood-mesenchymal stem cells in experimental acute kidney injury. Stem Cells. 2010;28(3):513–22. 10.1002/stem.293 . 20049901

[31] Du T, Cheng J, Zhong L, Zhao XF, Zhu J, Zhu YJ, et al The alleviation of acute and chronic kidney injury by human Wharton's jelly-derived mesenchymal stromal cells triggered by ischemia-reperfusion injury via an endocrine mechanism. Cytotherapy. 2012;14(10):1215–27. 10.3109/14653249.2012.711471 . 22920838

[32] Du T, Zou X, Cheng J, Wu S, Zhong L, Ju G, et al Human Wharton's jelly-derived mesenchymal stromal cells reduce renal fibrosis through induction of native and foreign hepatocyte growth factor synthesis in injured tubular epithelial cells. Stem Cell Res Ther. 2013;4(3):59 10.1186/scrt215 23734757 PMC3706832

[33] De Coppi P, Bartsch G Jr., Siddiqui MM, Xu T, Santos CC, Perin L, et al Isolation of amniotic stem cell lines with potential for therapy. Nat Biotechnol. 2007;25(1):100–6. 10.1038/nbt1274 . 17206138

[34] Mitka M. Amniotic cells show promise for fetal tissue engineering. JAMA: the journal of the American Medical Association. 2001;286(17):2083 . 11694128

[35] Kaviani A, Perry TE, Dzakovic A, Jennings RW, Ziegler MM, Fauza DO. The amniotic fluid as a source of cells for fetal tissue engineering. J Pediatr Surg. 2001;36(11):1662–5. . 11685697 10.1053/jpsu.2001.27945

[36] Prusa AR, Marton E, Rosner M, Bernaschek G, Hengstschlager M. Oct-4-expressing cells in human amniotic fluid: a new source for stem cell research? Hum Reprod. 2003;18(7):1489–93. Epub 2003/07/02. . 12832377 10.1093/humrep/deg279

[37] Prusa AR, Marton E, Rosner M, Bettelheim D, Lubec G, Pollack A, et al Neurogenic cells in human amniotic fluid. Am J Obstet Gynecol. 2004;191(1):309–14. Epub 2004/08/06. 10.1016/j.ajog.2003.12.014 S0002937803021392 [pii]. . 15295384

[38] Da Sacco S, Sedrakyan S, Boldrin F, Giuliani S, Parnigotto P, Habibian R, et al Human amniotic fluid as a potential new source of organ specific precursor cells for future regenerative medicine applications. J Urol. 2010;183(3):1193–200. Epub 2010/01/26. doi: S0022-5347(09)02892-4 [pii] 10.1016/j.juro.2009.11.006 20096867 PMC3174101

[39] Hauser PV, De Fazio R, Bruno S, Sdei S, Grange C, Bussolati B, et al Stem cells derived from human amniotic fluid contribute to acute kidney injury recovery. The American journal of pathology. 2010;177(4):2011–21. 10.2353/ajpath.2010.091245 20724594 PMC2947295

[40] Baulier E, Favreau F, Le Corf A, Jayle C, Schneider F, Goujon JM, et al Amniotic fluid-derived mesenchymal stem cells prevent fibrosis and preserve renal function in a preclinical porcine model of kidney transplantation. Stem cells translational medicine. 2014;3(7):809–20. 10.5966/sctm.2013-0186 24797827 PMC4073821

[41] Bieback K, Ha VA, Hecker A, Grassl M, Kinzebach S, Solz H, et al Altered gene expression in human adipose stem cells cultured with fetal bovine serum compared to human supplements. Tissue Eng Part A. 2010;16(11):3467–84. 10.1089/ten.TEA.2009.0727 . 20572797

[42] Da Sacco S, De Filippo RE, Perin L. Amniotic fluid as a source of pluripotent and multipotent stem cells for organ regeneration. Curr Opin Organ Transplant. 2011;16(1):101–5. 10.1097/MOT.0b013e3283424f6e . 21157345

[43] Da Sacco S, Lemley KV, Sedrakyan S, Zanusso I, Petrosyan A, Peti-Peterdi J, et al A novel source of cultured podocytes. PLoS One. 2013;8(12):e81812 10.1371/journal.pone.0081812 24349133 PMC3861313

[44] Zia S, Toelen J, Mori da Cunha M, Dekoninck P, de Coppi P, Deprest J. Routine clonal expansion of mesenchymal stem cells derived from amniotic fluid for perinatal applications. Prenatal diagnosis. 2013;33(10):921–8. 10.1002/pd.4162 . 23703584

[45] Morizane R, Monkawa T, Fujii S, Yamaguchi S, Homma K, Matsuzaki Y, et al Kidney specific protein-positive cells derived from embryonic stem cells reproduce tubular structures in vitro and differentiate into renal tubular cells. PLoS One. 2014;8(6):e64843 10.1371/journal.pone.0064843 23755150 PMC3670839

[46] Whyte DA, Li C, Thomson RB, Nix SL, Zanjani R, Karp SL, et al Ksp-cadherin gene promoter. I. Characterization and renal epithelial cell-specific activity. The American journal of physiology. 1999;277(4 Pt 2):F587–98. . 10516284 10.1152/ajprenal.1999.277.4.F587

[47] Bai Y, Pontoglio M, Hiesberger T, Sinclair AM, Igarashi P. Regulation of kidney-specific Ksp-cadherin gene promoter by hepatocyte nuclear factor-1beta. American journal of physiology Renal physiology. 2002;283(4):F839–51. 10.1152/ajprenal.00128.2002 . 12217876

[48] Monteiro Carvalho Mori da Cunha MG, Beckmann DV, Carlon MS, Zia S, Pippi NL, Mazzanti A, et al A surgical technique for homogenous renal distribution of substances in rats. European surgical research Europaische chirurgische Forschung Recherches chirurgicales europeennes. 2013;51(1–2):58–65. 10.1159/000354389 . 24081026

[49] Brady PD, Delle Chiaie B, Christenhusz G, Dierickx K, Van Den Bogaert K, Menten B, et al A prospective study of the clinical utility of prenatal chromosomal microarray analysis in fetuses with ultrasound abnormalities and an exploration of a framework for reporting unclassified variants and risk factors. Genetics in medicine: official journal of the American College of Medical Genetics. 2013 10.1038/gim.2013.168 . 24177055

[50] Arumugam TV, Shiels IA, Strachan AJ, Abbenante G, Fairlie DP, Taylor SM. A small molecule C5a receptor antagonist protects kidneys from ischemia/reperfusion injury in rats. Kidney international. 2003;63(1):134–42. 10.1046/j.1523-1755.2003.00737.x . 12472776

[51] Turgut F, Bayrak O, Catal F, Bayrak R, Atmaca AF, Koc A, et al Antioxidant and protective effects of silymarin on ischemia and reperfusion injury in the kidney tissues of rats. International urology and nephrology. 2008;40(2):453–60. 10.1007/s11255-008-9365-4 . 18368506

[52] Bayrak O, Uz E, Bayrak R, Turgut F, Atmaca AF, Sahin S, et al Curcumin protects against ischemia/reperfusion injury in rat kidneys. World journal of urology. 2008;26(3):285–91. 10.1007/s00345-008-0253-4 . 18373094

[53] Liu M, Liang Y, Chigurupati S, Lathia JD, Pletnikov M, Sun Z, et al Acute kidney injury leads to inflammation and functional changes in the brain. Journal of the American Society of Nephrology: JASN. 2008;19(7):1360–70. 10.1681/ASN.2007080901 18385426 PMC2440297

[54] Heukeshoven J, Dernick R. Characterization of a solvent system for separation of water-insoluble poliovirus proteins by reversed-phase high-performance liquid chromatography. Journal of chromatography. 1985;326:91–101. . 2993331 10.1016/s0021-9673(01)87434-3

[55] Sekiya I, Vuoristo JT, Larson BL, Prockop DJ. In vitro cartilage formation by human adult stem cells from bone marrow stroma defines the sequence of cellular and molecular events during chondrogenesis. Proc Natl Acad Sci U S A. 2002;99(7):4397–402. 10.1073/pnas.052716199 11917104 PMC123659

[56] Duplomb L, Dagouassat M, Jourdon P, Heymann D. Concise review: embryonic stem cells: a new tool to study osteoblast and osteoclast differentiation. Stem Cells. 2007;25(3):544–52. 10.1634/stemcells.2006-0395 . 17095705

[57] Dobreva MP, Pereira PN, Deprest J, Zwijsen A. On the origin of amniotic stem cells: of mice and men. The International journal of developmental biology. 2010;54(5):761–77. 10.1387/ijdb.092935md . 20446274

[58] Kreidberg JA. WT1 and kidney progenitor cells. Organogenesis. 2010;6(2):61–70. 20885852 10.4161/org.6.2.11928 PMC2901809

[59] Gilbert WM, Brace RA. Amniotic fluid volume and normal flows to and from the amniotic cavity. Seminars in perinatology. 1993;17(3):150–7. . 8378799

[60] Underwood MA, Gilbert WM, Sherman MP. Amniotic fluid: not just fetal urine anymore. Journal of perinatology: official journal of the California Perinatal Association. 2005;25(5):341–8. 10.1038/sj.jp.7211290 . 15861199

[61] Li P, Zhang R, Sun H, Chen L, Liu F, Yao C, et al PKH26 can transfer to host cells in vitro and vivo. Stem Cells Dev. 2013;22(2):340–4. 10.1089/scd.2012.0357 22913652 PMC3545314

[62] Lassailly F, Griessinger E, Bonnet D. "Microenvironmental contaminations" induced by fluorescent lipophilic dyes used for noninvasive in vitro and in vivo cell tracking. Blood. 2010;115(26):5347–54. 10.1182/blood-2009-05-224030 . 20215639

[63] Griffin MD, Ritter T, Mahon BP. Immunological aspects of allogeneic mesenchymal stem cell therapies. Hum Gene Ther. 2010;21(12):1641–55. 10.1089/hum.2010.156 . 20718666

[64] Glenn JD, Whartenby KA. Mesenchymal stem cells: Emerging mechanisms of immunomodulation and therapy. World journal of stem cells. 2014;6(5):526–39. 10.4252/wjsc.v6.i5.526 25426250 PMC4178253

[65] Togel F, Yang Y, Zhang P, Hu Z, Westenfelder C. Bioluminescence imaging to monitor the in vivo distribution of administered mesenchymal stem cells in acute kidney injury. American journal of physiology Renal physiology. 2008;295(1):F315–21. 10.1152/ajprenal.00098.2008 18480180 PMC4063418

[66] Zonta S, De Martino M, Bedino G, Piotti G, Rampino T, Gregorini M, et al Which is the most suitable and effective route of administration for mesenchymal stem cell-based immunomodulation therapy in experimental kidney transplantation: endovenous or arterial? Transplant Proc. 2010;42(4):1336–40. 10.1016/j.transproceed.2010.03.081 . 20534295

[67] Cai J, Yu X, Xu R, Fang Y, Qian X, Liu S, et al Maximum efficacy of mesenchymal stem cells in rat model of renal ischemia-reperfusion injury: renal artery administration with optimal numbers. PloS one. 2014;9(3):e92347 10.1371/journal.pone.0092347 24637784 PMC3956922

[68] Saenz-Morales D, Conde E, Blanco-Sanchez I, Ponte B, Aguado-Fraile E, de Las Casas G, et al Differential resolution of inflammation and recovery after renal ischemia-reperfusion injury in Brown Norway compared with Sprague Dawley rats. Kidney international. 2010;77(9):781–93. 10.1038/ki.2010.10 . 20164827

[69] Humphreys BD, Bonventre JV. Mesenchymal stem cells in acute kidney injury. Annual review of medicine. 2008;59:311–25. 10.1146/annurev.med.59.061506.154239 . 17914926

[70] Geng Y, Zhang L, Fu B, Zhang J, Hong Q, Hu J, et al Mesenchymal stem cells ameliorate rhabdomyolysis-induced acute kidney injury via the activation of M2 macrophages. Stem cell research & therapy. 2014;5(3):80 10.1186/scrt469 24961539 PMC4230233

[71] Ricardo SD, van Goor H, Eddy AA. Macrophage diversity in renal injury and repair. The Journal of clinical investigation. 2008;118(11):3522–30. 10.1172/JCI36150 18982158 PMC2575702

[72] Lee S, Huen S, Nishio H, Nishio S, Lee HK, Choi BS, et al Distinct macrophage phenotypes contribute to kidney injury and repair. Journal of the American Society of Nephrology: JASN. 2011;22(2):317–26. 10.1681/ASN.2009060615 21289217 PMC3029904

[73] LeBleu VS, Taduri G, O'Connell J, Teng Y, Cooke VG, Woda C, et al Origin and function of myofibroblasts in kidney fibrosis. Nat Med. 2013;19(8):1047–53. 10.1038/nm.3218 23817022 PMC4067127

[74] Krolewski AS, Niewczas MA, Skupien J, Gohda T, Smiles A, Eckfeldt JH, et al Early progressive renal decline precedes the onset of microalbuminuria and its progression to macroalbuminuria. Diabetes care. 2014;37(1):226–34. 10.2337/dc13-0985 23939543 PMC3867993

[75] Li L, Truong P, Igarashi P, Lin F. Renal and bone marrow cells fuse after renal ischemic injury. Journal of the American Society of Nephrology: JASN. 2007;18(12):3067–77. 10.1681/ASN.2007030284 . 18003777

[76] Yamashita T, Fujimiya M, Nagaishi K, Ataka K, Tanaka M, Yoshida H, et al Fusion of bone marrow-derived cells with renal tubules contributes to renal dysfunction in diabetic nephropathy. FASEB journal: official publication of the Federation of American Societies for Experimental Biology. 2012;26(4):1559–68. 10.1096/fj.11-183194 . 22198389

[77] Lam AQ, Freedman BS, Morizane R, Lerou PH, Valerius MT, Bonventre JV. Rapid and efficient differentiation of human pluripotent stem cells into intermediate mesoderm that forms tubules expressing kidney proximal tubular markers. Journal of the American Society of Nephrology: JASN. 2014;25(6):1211–25. 10.1681/ASN.2013080831 24357672 PMC4033376

[78] Zarjou A, Kim J, Traylor AM, Sanders PW, Balla J, Agarwal A, et al Paracrine effects of mesenchymal stem cells in cisplatin-induced renal injury require heme oxygenase-1. American journal of physiology Renal physiology. 2011;300(1):F254–62. 10.1152/ajprenal.00594.2010 21048024 PMC3023217

[79] Bonventre JV. Microvesicles from mesenchymal stromal cells protect against acute kidney injury. Journal of the American Society of Nephrology: JASN. 2009;20(5):927–8. 10.1681/ASN.2009030322 . 19389839

[80] Reis LA, Borges FT, Simoes MJ, Borges AA, Sinigaglia-Coimbra R, Schor N. Bone marrow-derived mesenchymal stem cells repaired but did not prevent gentamicin-induced acute kidney injury through paracrine effects in rats. PLoS One. 2012;7(9):e44092 10.1371/journal.pone.0044092 22970165 PMC3435420

[81] Moncada S. Nitric oxide in the vasculature: physiology and pathophysiology. Annals of the New York Academy of Sciences. 1997;811:60–7; discussion 7–9. . 9186585 10.1111/j.1749-6632.1997.tb51989.x

