# Supplementary figures and images for: Amniotic Fluid Derived Stem Cells with a Renal Progenitor Phenotype Inhibit Interstitial Fibrosis in Renal Ischemia and Reperfusion Injury in Rats
Source: PLoS One. 2015 Aug 21;10(8):e0136145. doi: 10.1371/journal.pone.0136145 (PMC4546614; doi:10.1371/journal.pone.0136145)

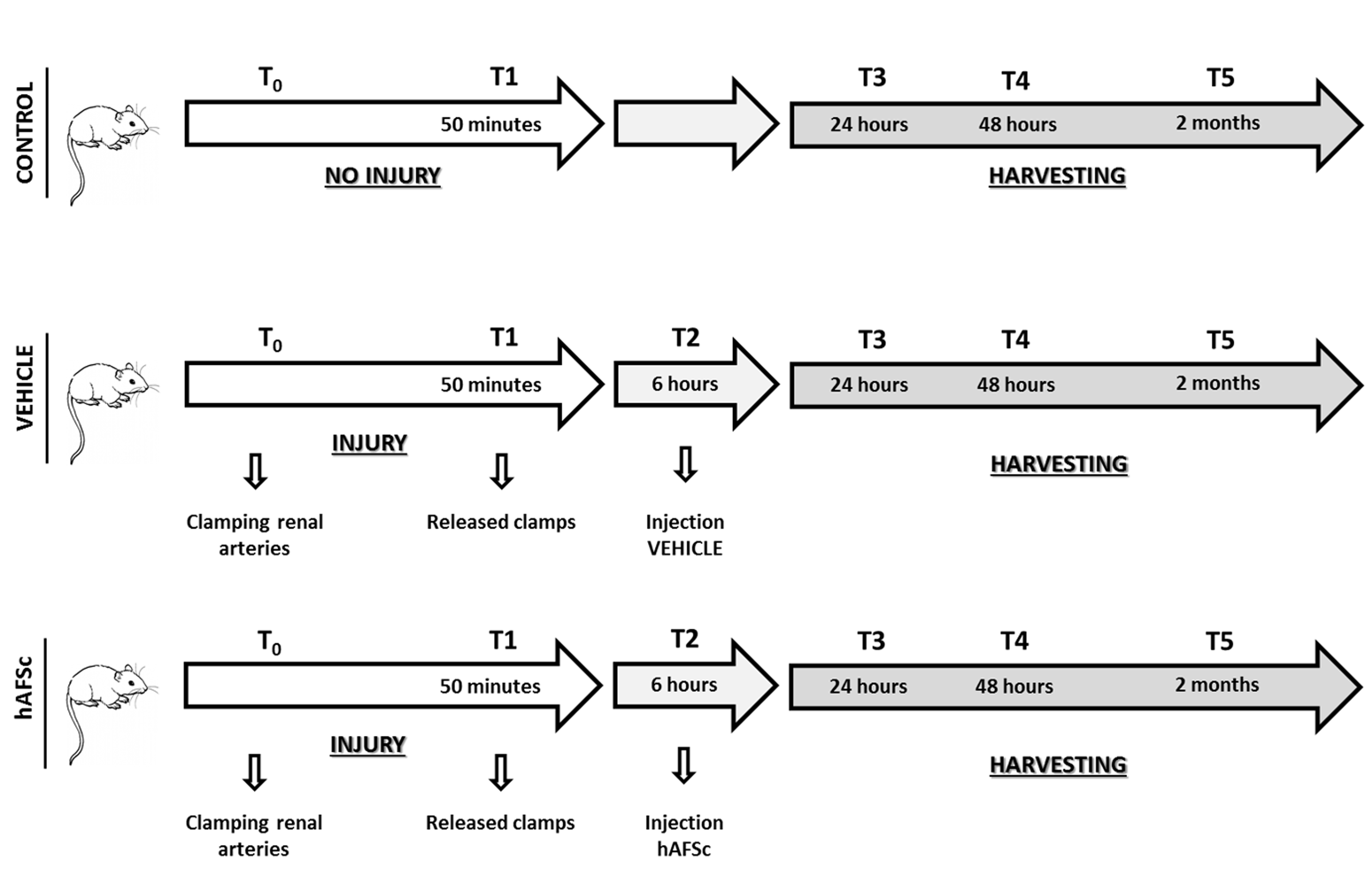

Supplement: S1 Fig — (TIF) [file pone.0136145.s001.tif]

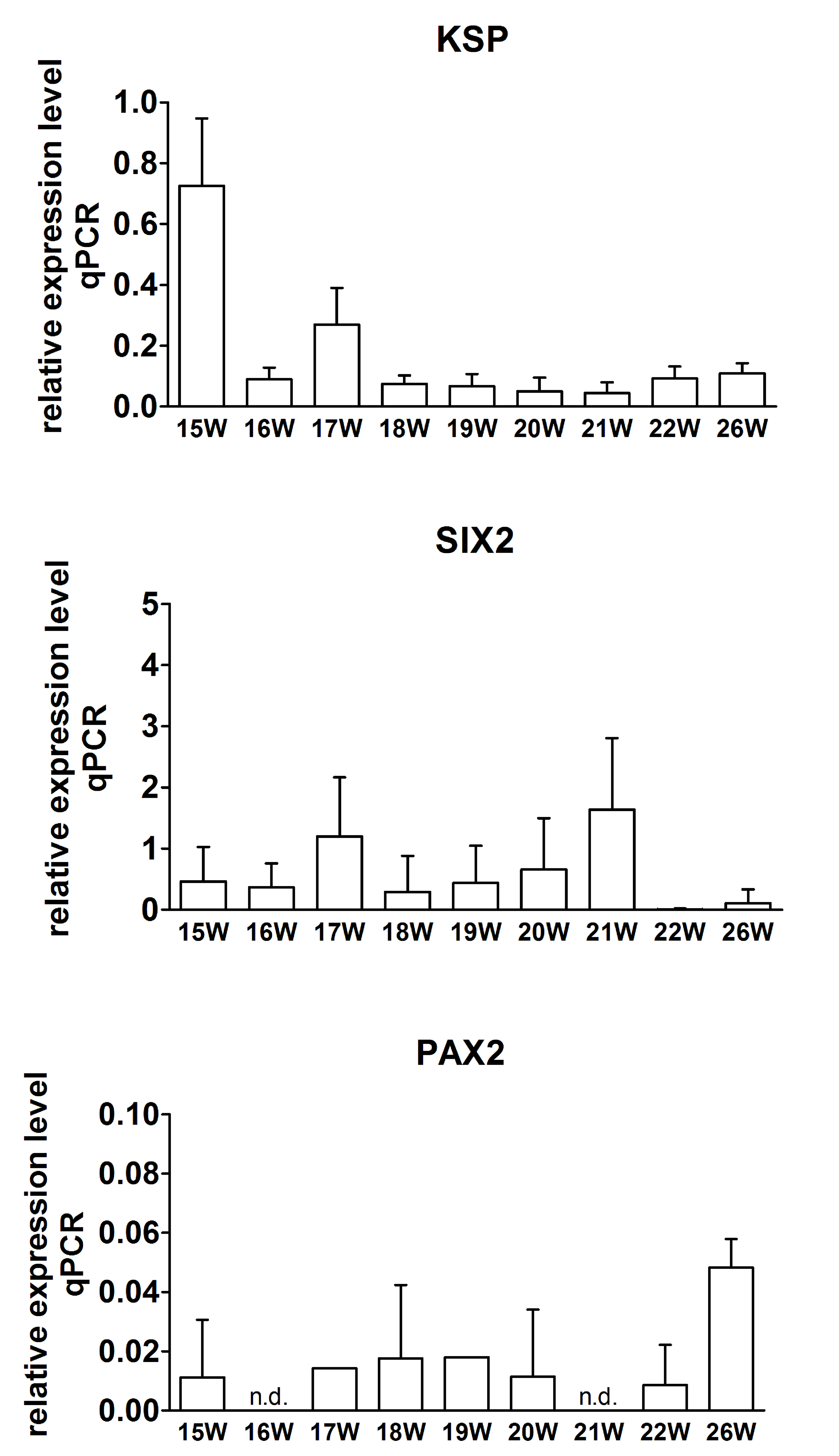

Supplement: S2 Fig — Expression level is calculated to the expression of these markers in PTEC cell line. (TIF) [file pone.0136145.s002.tif]

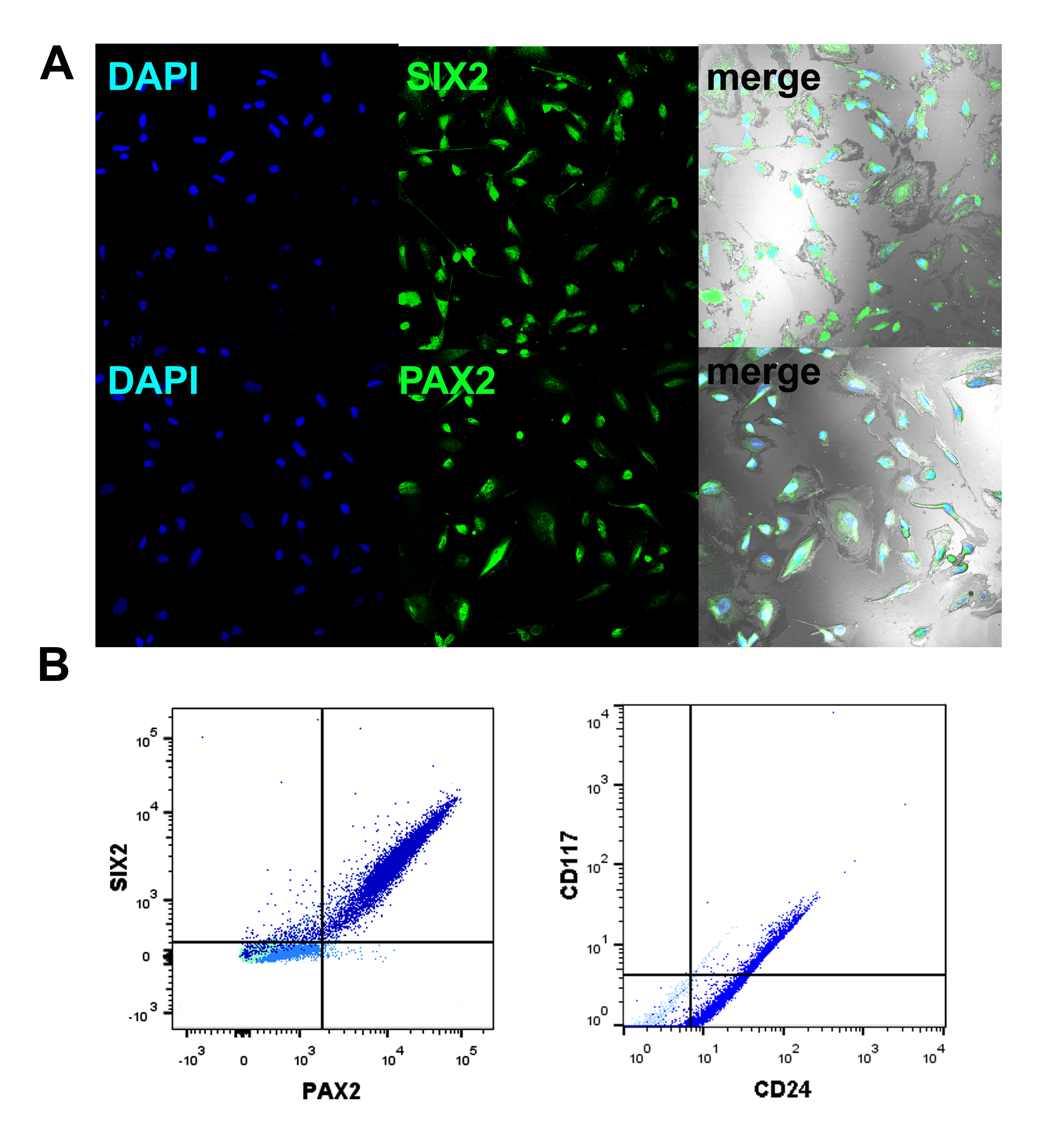

Supplement: S3 Fig — (TIF) [file pone.0136145.s003.tif]

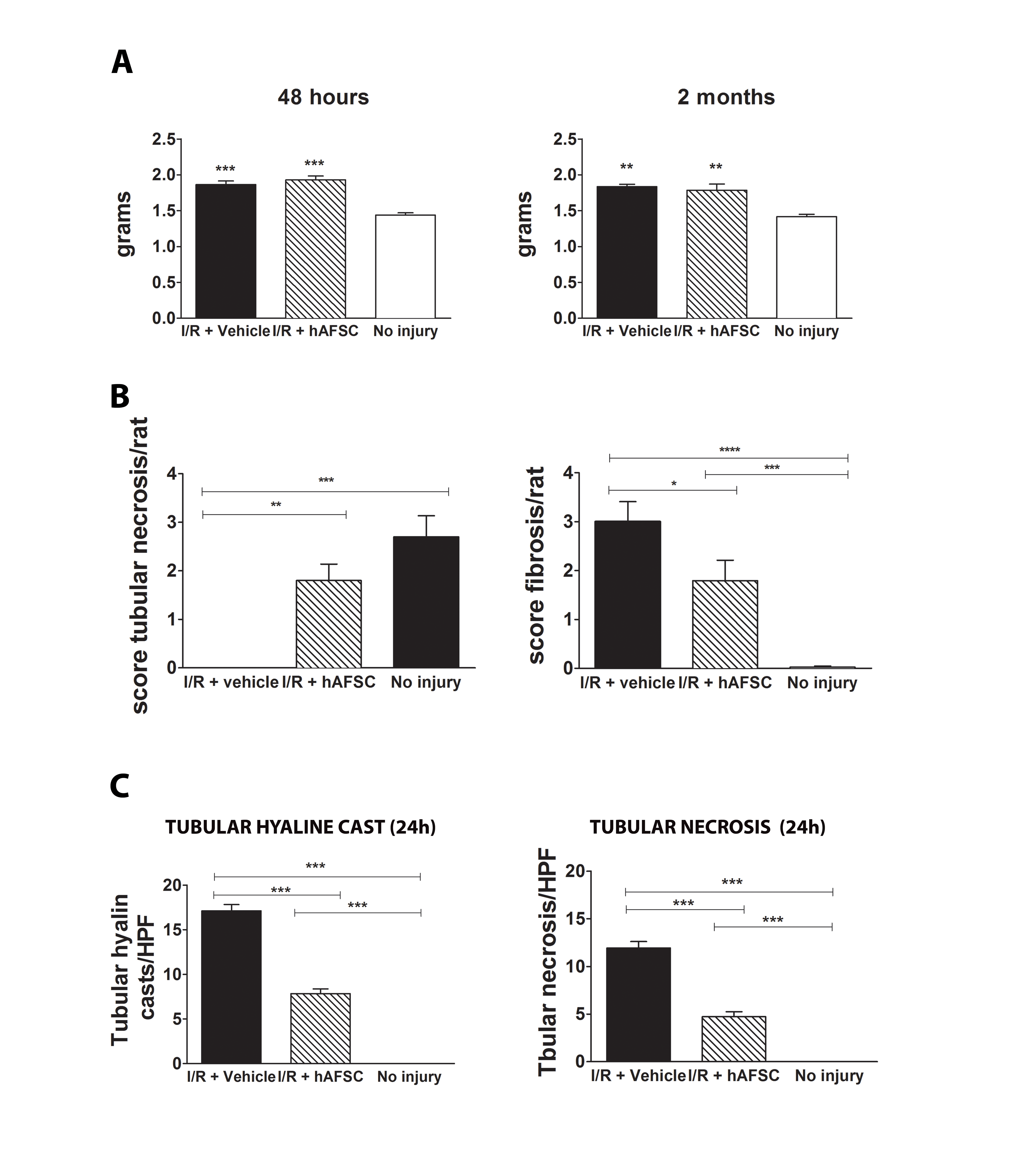

Supplement: S4 Fig — (TIF) [file pone.0136145.s004.tif]

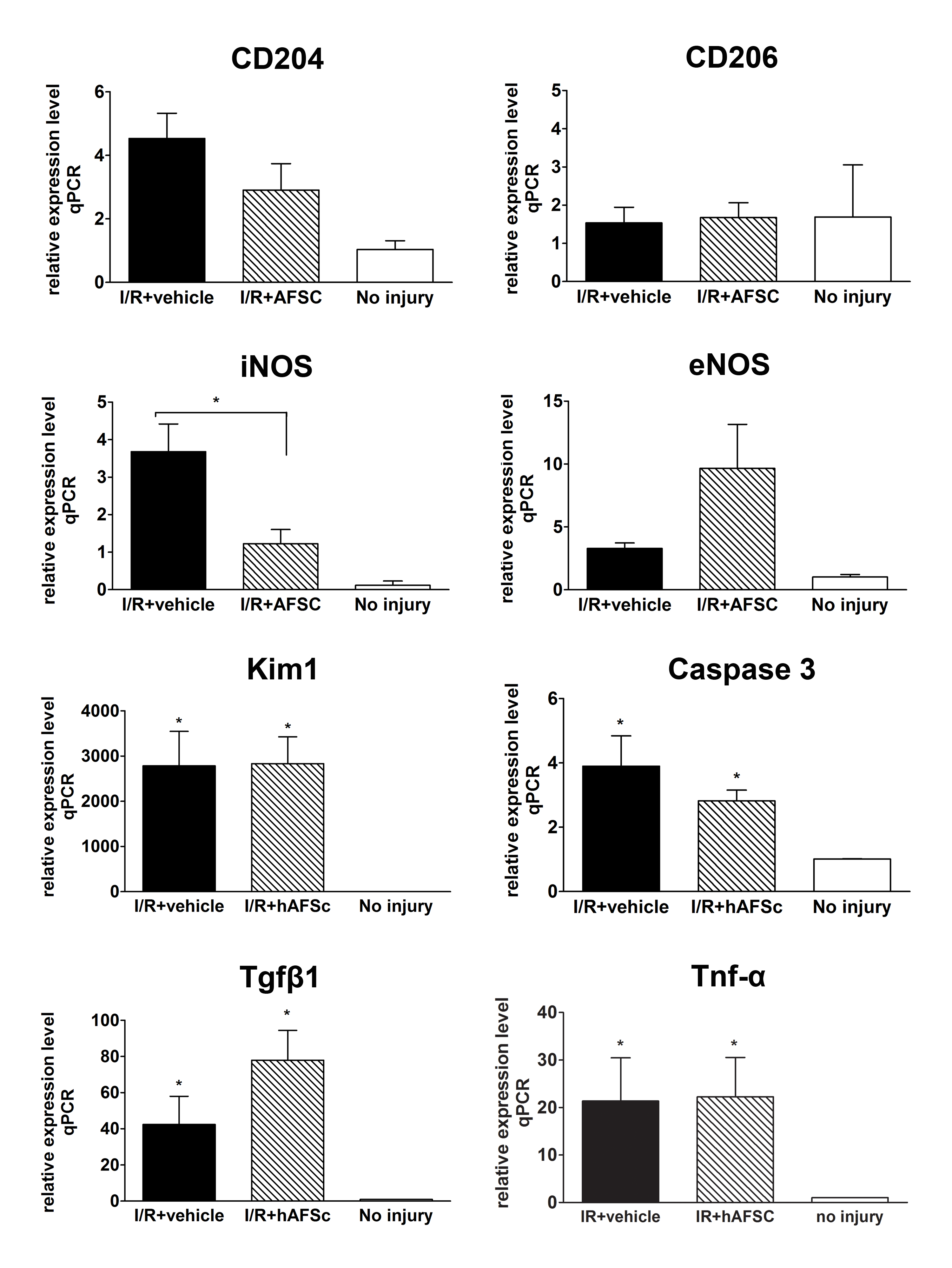

Supplement: S5 Fig — Expression level of the markers CD204, CD206, iNOS, eNOS, Kim1, Caspase3, TGFβ1 and TNFα in the three groups. * p<0.05. (TIF) [file pone.0136145.s005.tif]
